# Supplementary material for: Exploring the science and data foundation for Federal public lands decisions
Source: PLoS One. 2025 Feb 10;20(2):e0316013. doi: 10.1371/journal.pone.0316013 (PMC11809896; doi:10.1371/journal.pone.0316013)
Supplement: S1 File — (PDF) [file pone.0316013.s001.pdf]

Any use of trade, firm, or product names is for descriptive purposes only and does not imply endorsement by the U.S. Government.

Supporting Information Item 1.

Decision tree:
Use this decision tree to determine the type of document cited in the Environmental Assessment. Select only one document type for each citation. For documents where the author or document type is not clear, spend up to, but no more than, five minutes looking for the document using the Google search engine. Document types that are categorized as journal articles, Bureau of Land Management (BLM) land use plans, and National Environmental Policy Act (NEPA) documents do not need to be looked up using Google if the document type is clear based on the information in the References cited section of the Environmental Assessment.

1. *Is it a* **journal article?** [*If not, continue moving down the list here and for all items below]* If so, confirm that it is from a legitimate journal (e.g., not from a journal considered to be a predatory, open access journal such as the ones described here: https://library.medicine.yale.edu/scholarly-communication/predatory-publishing).

2. *If not a journal article, is it a* **law, policy, manual, handbook, BLM technical reference, or guidance document?** These are any document that provides guidance on how to do something. The document may or may not be authored by BLM. These documents provide guidance to BLM or state a rule that BLM must follow (e.g., Endangered Species Act , Clean Water Act). Almost all documents that fall in this category will have a number or code, with the exception of some handbooks or guidance documents. Examples of documents in this category include laws (e.g., citations of the Endangered Species Act or Clean Water Act), statutes, administrative code, manuals, or handbooks from the Department of the Interior (DOI), BLM (e.g., BLM's NEPA or land use planning handbook, a BLM Technical Note), or another agency; BLM Instructional Memos (IMs); BLM Information Bulletins (IBs); DOI Secretarial Orders; and Executive Orders.

3. *If not one of the document types above, is it a* **science or monitoring plan or strategy?** The document may or may not have been prepared by BLM (e.g., the U.S. Geological Survey (USGS) wrote a science plan about wild horses for BLM). Science or monitoring plans or strategies include national level strategies, plans for national monuments or National Conservation Areas (NCAs), recovery plans for specific listed species (most likely developed by U.S. Fish and Wildlife Service), and monitoring strategies, plans, or frameworks (e.g., related to greater sage-grouse or BLM's Assessment, Inventory, and Monitoring (AIM) program).

4. *If not one of the document types above, is it a* **plan to manage a resource, land use plan, or NEPA document?** This category includes NEPA documents (e.g., Environmental Impact Statements, Environmental Assessments, Determinations of NEPA Adequacy, Categorical Exclusions, reasonably foreseeable development

scenarios), BLM Resource Management Plans, Biological Opinions, Biological Assessments, species conservation strategies, and appendices or addendums to any of the previously mentioned documents.

4a. Is it **authored by the BLM**?

4b. Is it **not authored by the BLM**? Examples of these documents include a land use plan completed by the U.S. Forest Service or a Biological Opinion completed by the U.S. Fish and Wildlife Service.

5. *If not one of the document types above, is it an* **inventory or dataset?** This would include any collection of data or any inventory of a resource, including maps (as long as they include data on the resource of concern) and cultural resource inventories. Note that the data do not need to be an excel file or collection of values to qualify. For example, a description of soils in the project area is considered an inventory. Additionally, a report that contains ONLY data falls into this category [Note: A map that only contains information on the location of the project does not qualify as a data or science citation and thus the map citation should not be coded.]

Further,

5a. *Is it* **published or readily available**? The document should have a product name, number, or digital object identifier to fall in this category. To be considered published or publicly available, you must be able to be find the document within five minutes using the Google search engine when searching using the citation or digital object identifier, or it must be available to the public by request or for a small fee.

5a. i. *Is it* **authored by the BLM?** BLM-authored published or readily available inventories and datasets include BLM AIM data.

5a. ii. *Is it* **not authored by the BLM?** Non-BLM published or readily available inventories or datasets include products such as data produced by the Landscape Fire and Resource Management Planning Tools Program (LANDFIRE), soils data produced by the U.S. Department of Agriculture Natural Resources Conservation Service, landcover data produced by the Multi-Resolution Land Characteristics Consortium, and USGS Digital Data Series.

5b. *Is it* **unpublished or not readily available**? Use this category if the document lacks the information stated above for published or readily available inventories or datasets, or cannot be found using Google within five minutes. A map falls into this category; however, it must be a map that includes data about a specific resource (such as the location of a rare plant). A map that only shows the location of the project is not considered a science or data reference. Any inventory or dataset citation with a broken link falls into this category, as it is not readily available.

5b. i. *Is it* **authored by the BLM?** BLM-authored and unpublished or not publicly available inventories and datasets in this category include BLM reports completed by a field office where the title of the report indicates that it is an inventory of a resource (e.g., cultural resources, rare plants, invasive species).

5b. ii. *Is it* **not authored by the BLM?** Non-BLM and unpublished or not publicly available inventories or datasets include cultural resource or other types of inventories completed and authored by an entity other than the BLM (e.g., an external contractor).

6. *If not one of the document types above, is it a* **report?** A report is a document that presents information in an organized format for a specific audience and purpose. A report will likely contain some data, but it does not entirely consist of data. If the document consists only of data, it should be classified into the inventory or data category.

Further,

6a. *Is it a* **peer-reviewed science report**? This category includes peer-reviewed, published reports of science or research – information that is unbiased, objective, and peer-reviewed. It may present original science findings or summaries or syntheses of science findings (e.g., a Biological Assessment), sometimes for non-science audiences (e.g., policy makers). If the report is not new science, it must be commissioned or peer-reviewed by an authority on the subject (e.g., Intergovernmental Panel on Climate Change, USGS, U.S. Forest Service (USFS) Research Station).

To classify the document as a peer-reviewed science report, you need to be able to answer the following questions: *Is it clear…*

… that it has a digital object identifier OR is peer-reviewed?
… who the publisher is?
… who the author is?
… when it was published?
… that there is a report number? [may be missing for non-agency reports]
… that it is a specific type of report (e.g., technical report, USGS Open-File Report)? [may be missing for non-agency reports]

6a. i. *Is it a* **peer-reviewed science report authored by the BLM?** BLM-published reports will include a report number and will look like they are edited and published (e.g., BLM technical reports). Note that BLM Technical References fall under category #2 (law, policy, manual, handbook, BLM technical reference, or guidance document).

6a. ii. *Is it a* **peer-reviewed science report not authored by the BLM?** Non-BLM authored published reports include USGS Open-File Reports, Scientific Investigator Reports, and Bulletins; USFS General Technical Reports, and reports from a college or university.

6b. *Is it a* **other report**? Reports for which you cannot respond 'yes' to all of the questions in 6a or for which you are not sure that they went through a peer-review process. This category includes reports that contain science or research results but do not meet the criteria above (e.g., study reports prepared by a contractor). Most reports that are not from government agencies will fall in this category. Any report citation for which the link is broken falls into this category, as it is not readily available.

6b. i. *Is it a* **other report authored by the BLM?** BLM unpublished reports will include a report number and are often written by field

offices. This category includes Allotment Master Reports written by the BLM.

6b. ii. *Is it a* **other report not authored by the BLM?** Unpublished reports not authored by BLM often include reports prepared for the BLM by an external contractor.

7. *If not one of the document types above, is it a* **website?** This category includes websites that are not reports and websites that do not provide data.

8. *If not one of the document types above, is it a* **other science product.** This category includes books, newspapers, personal communications, formally cited emails, any articles from non-reputable journals, theses, dissertations, conference papers/proceedings/abstracts/reports; or other miscellaneous documents such as permit applications. Also, any citations lacking information to determine the document type fall in this category.

9. *If not one of the document types above and you are unsure how to categorize the document, classify it as* **unsure.**
