# Supplementary material for: Exploring the science and data foundation for Federal public lands decisions
Source: PLoS One. 2025 Feb 10;20(2):e0316013. doi: 10.1371/journal.pone.0316013 (PMC11809896; doi:10.1371/journal.pone.0316013)
Supplement: S2 File — This file contains figures of the age of citation and type of documents cited for all resources that 1) were analyzed five or more times across all Environmental Assessments and 2) had a total of at least ten citations across all analysis sections for that resource: Air quality and climate (S1 Fig); Aquatic wildlife (S2 Fig); Archaeological and historic resources (S3 Fig); Fire ecology and management (S4 Fig); Geology (S5 Fig); Grazing and range (S6 Fig); Invasive plants (S7 Fig); Mineral resources (S8 Fig); Noise (S9 Fig); Protected birds (S10 Fig); Recreation (S11 Fig); Sage-grouse (S12 Fig); Socioeconomics (S13 Fig); Soils (S14 Fig); Sensitive aquatic wildlife (S15 Fig); Sensitive plants (S16 Fig); Sensitive terrestrial wildlife (S17 Fig); Terrestrial wildlife (S18 Fig); Vegetation (S19 Fig); Visual resources (S20 Fig); Water (S21 Fig); Wetlands and riparian areas (S22 Fig); Wild horses and burros (S23 Fig). (PDF) [file pone.0316013.s002.pdf]

**Supporting Figures**

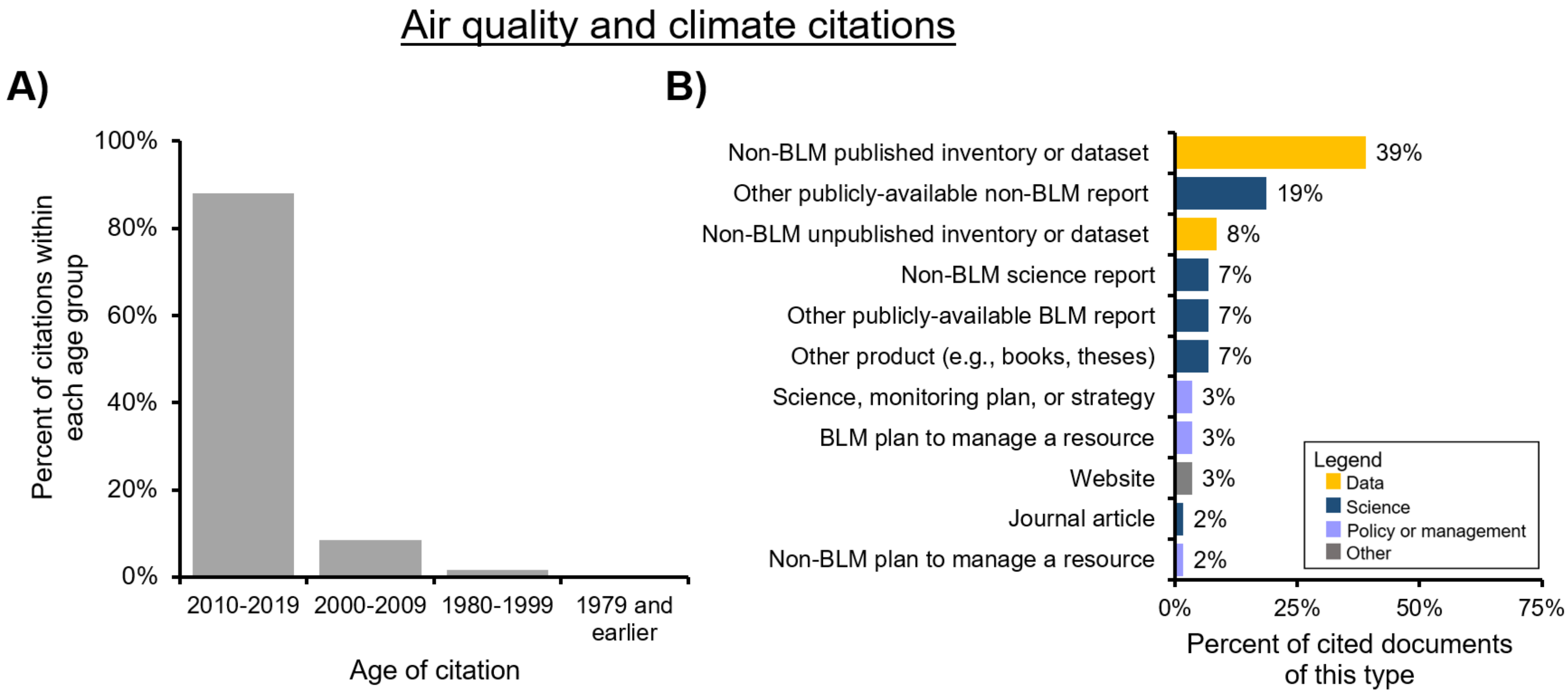


**Figure S1. Air quality and climate citations in Bureau of Land Management (BLM) Environmental Assessments.** A) Age of citations and B) types of documents cited in air quality and climate resource analysis sections (59 total citations). Two citations of unknown age are not included in figure A. The documents presented are from a stratified random sample of 70 Environmental Assessments completed by the BLM in Colorado from 2015-2019.

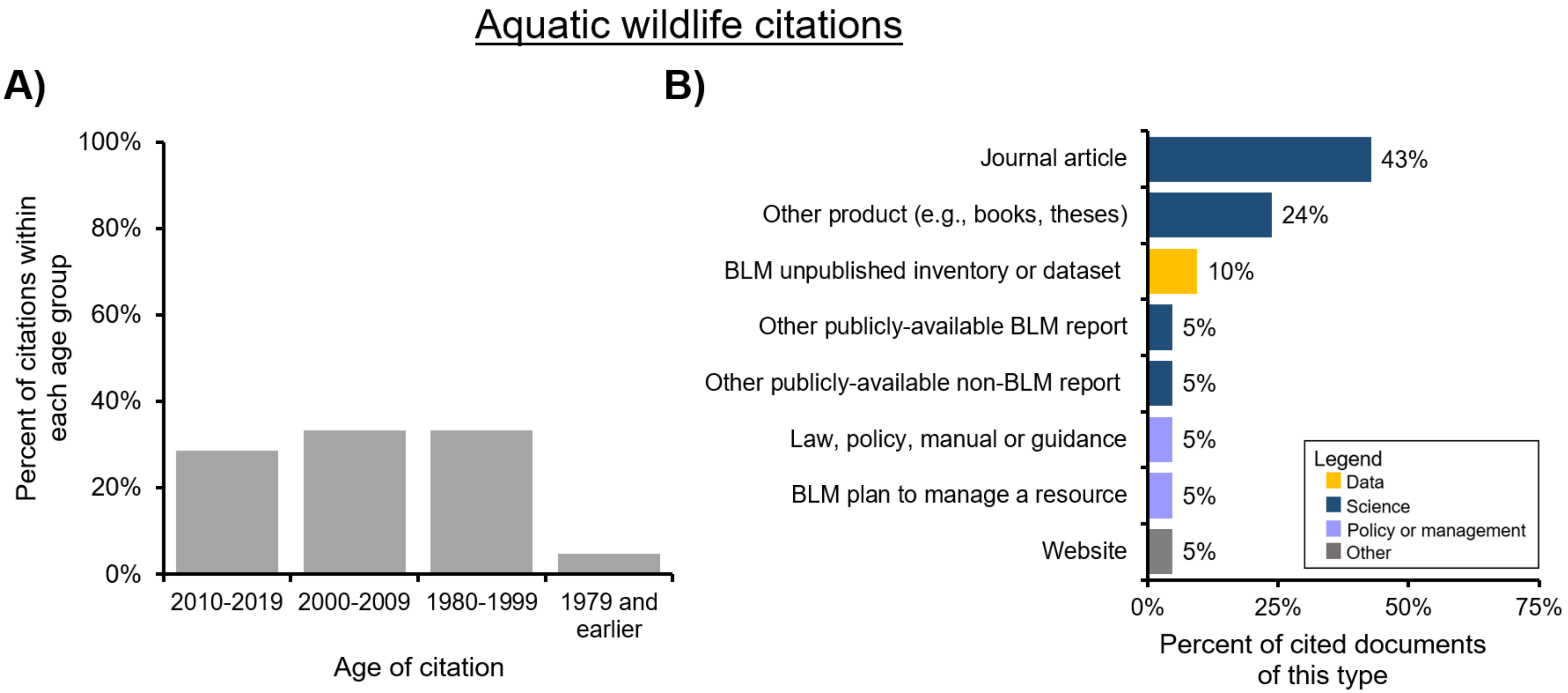


**Figure S2. Aquatic wildlife citations in Bureau of Land Management (BLM) Environmental Assessments.** A) Age of citations and B) types of documents cited in aquatic wildlife resource analysis sections (21 total citations). The documents presented are from a stratified random sample of 70 Environmental Assessments completed by the BLM in Colorado from 2015-2019.

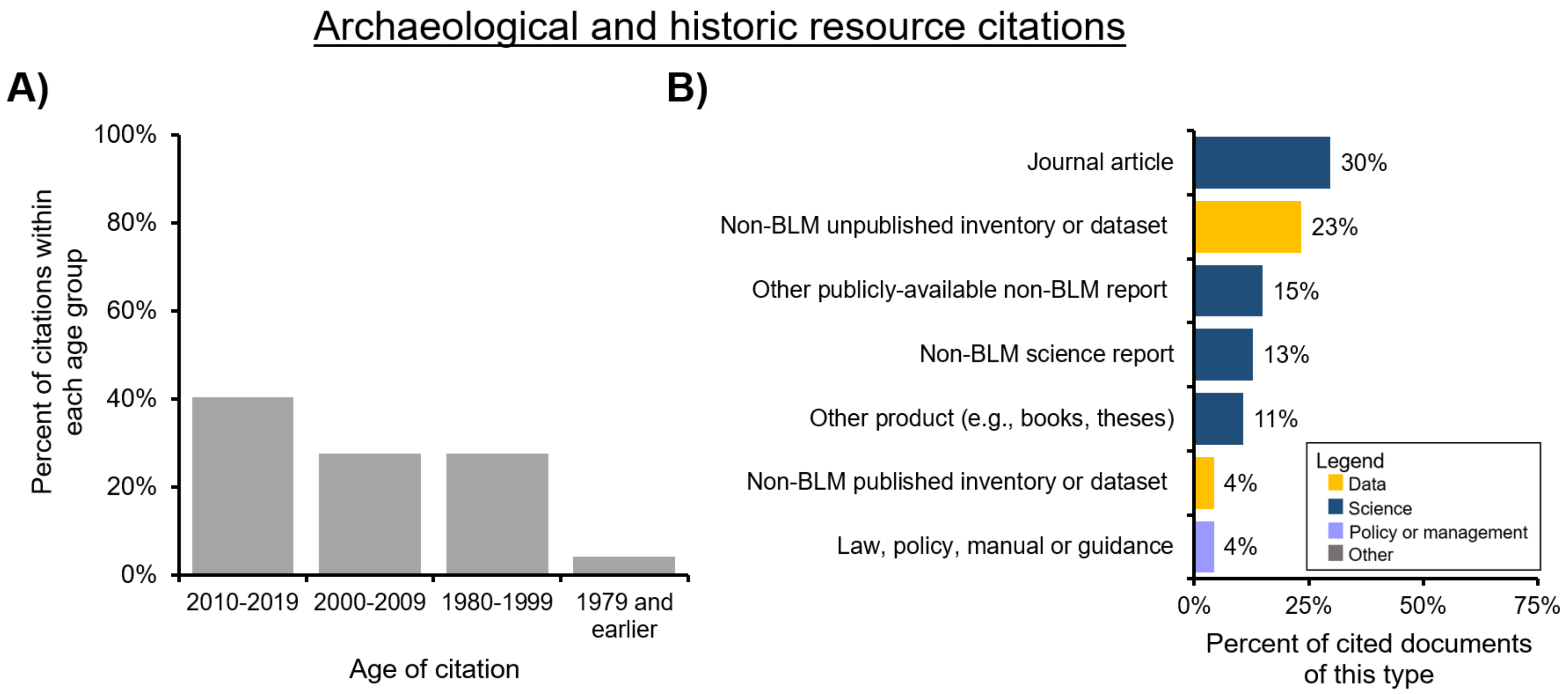


**Figure S3. Archaeological and historic resource citations in Bureau of Land Management (BLM) Environmental Assessments.** A) Age of citations and B) types of documents cited in archaelological and historic resource analysis sections (47 total citations). The documents presented are from a stratified random sample of 70 Environmental Assessments completed by the BLM in Colorado from 2015-2019.

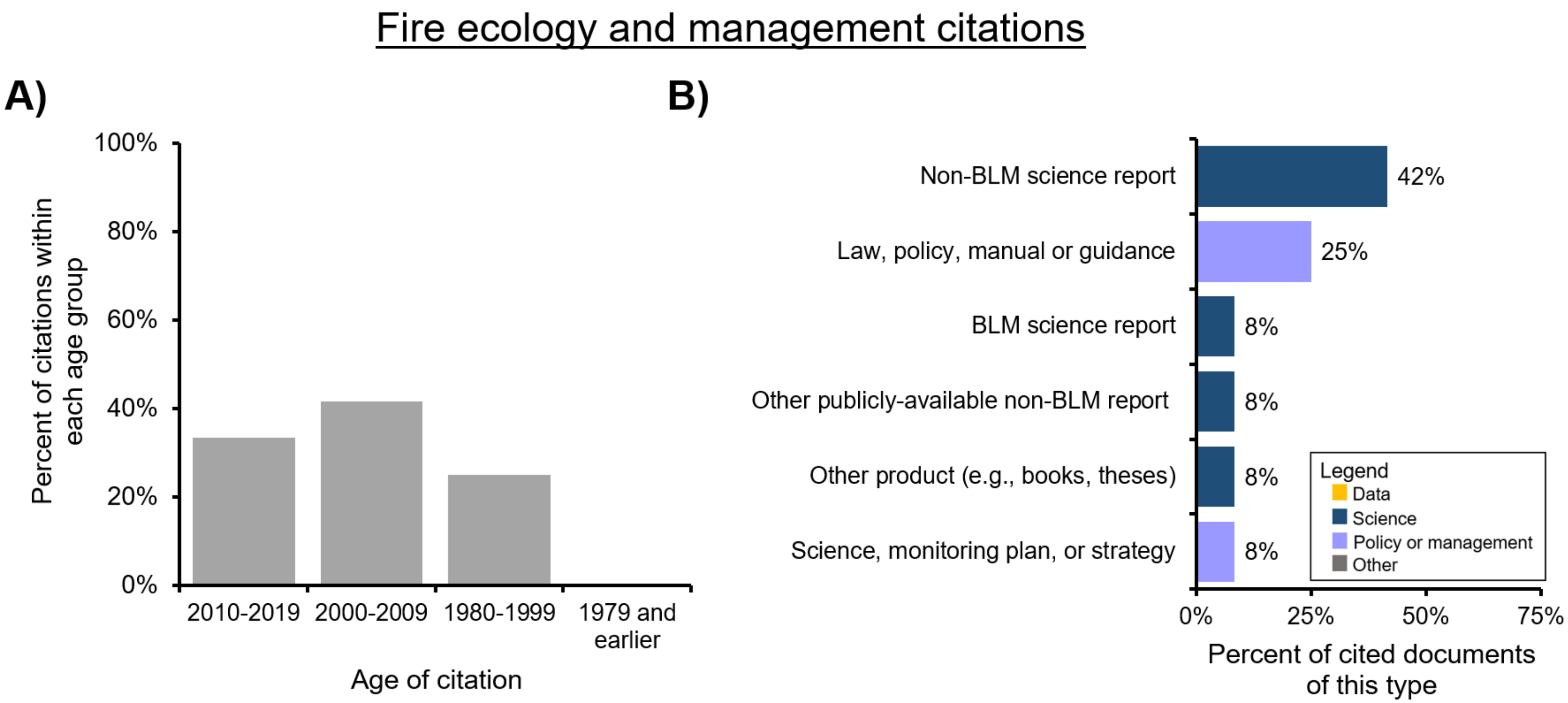


**Figure S4. Fire ecology and management citations in Bureau of Land Management (BLM) Environmental Assessments.** A) Age of citations and B) types of documents cited in fire ecology and management resource analysis sections (12 total citations). The documents presented are from a stratified random sample of 70 Environmental Assessments completed by the BLM in Colorado from 2015-2019.

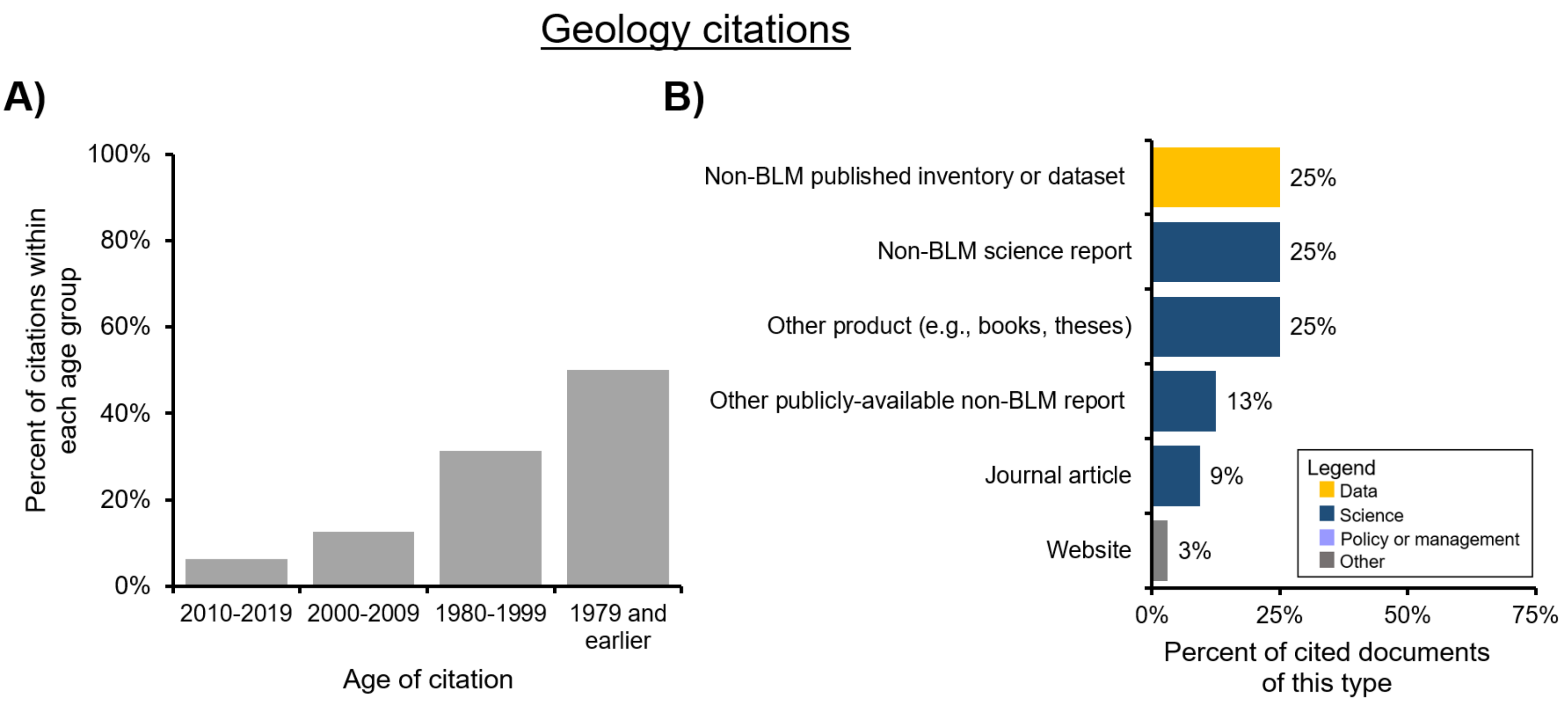


**Figure S5. Geology citations in Bureau of Land Management (BLM) Environmental Assessments.** A) Age of citations and B) types of documents cited in geology resource analysis sections (32 total citations). The documents presented are from a stratified random sample of 70 Environmental Assessments completed by the BLM in Colorado from 2015-2019.

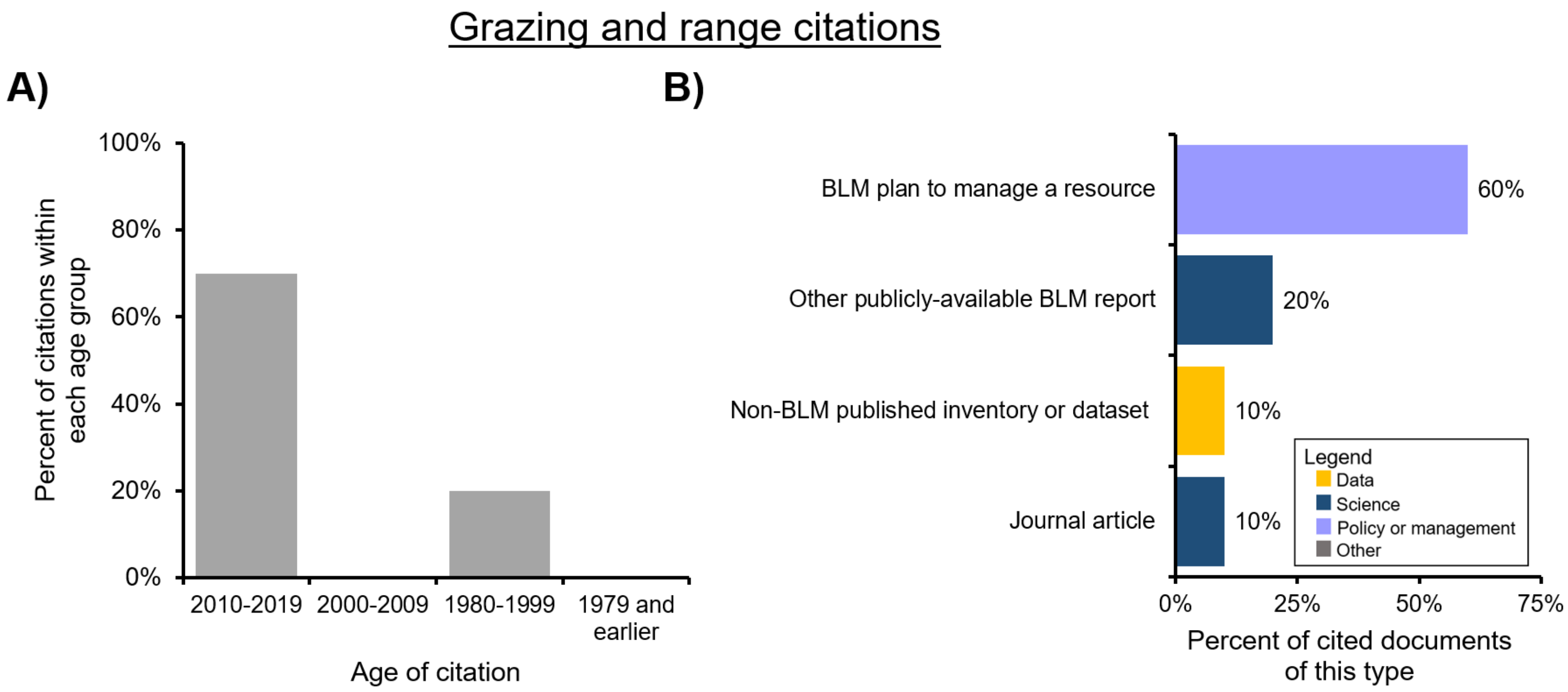


**Figure S6. Grazing and range citations in Bureau of Land Management (BLM) Environmental Assessments.** A) Age of citations and B) types of documents cited in grazing and range resource analysis sections (10 total citations). One citation of unknown age is not included in figure A. The documents presented are from a stratified random sample of 70 Environmental Assessments completed by the BLM in Colorado from 2015-2019.

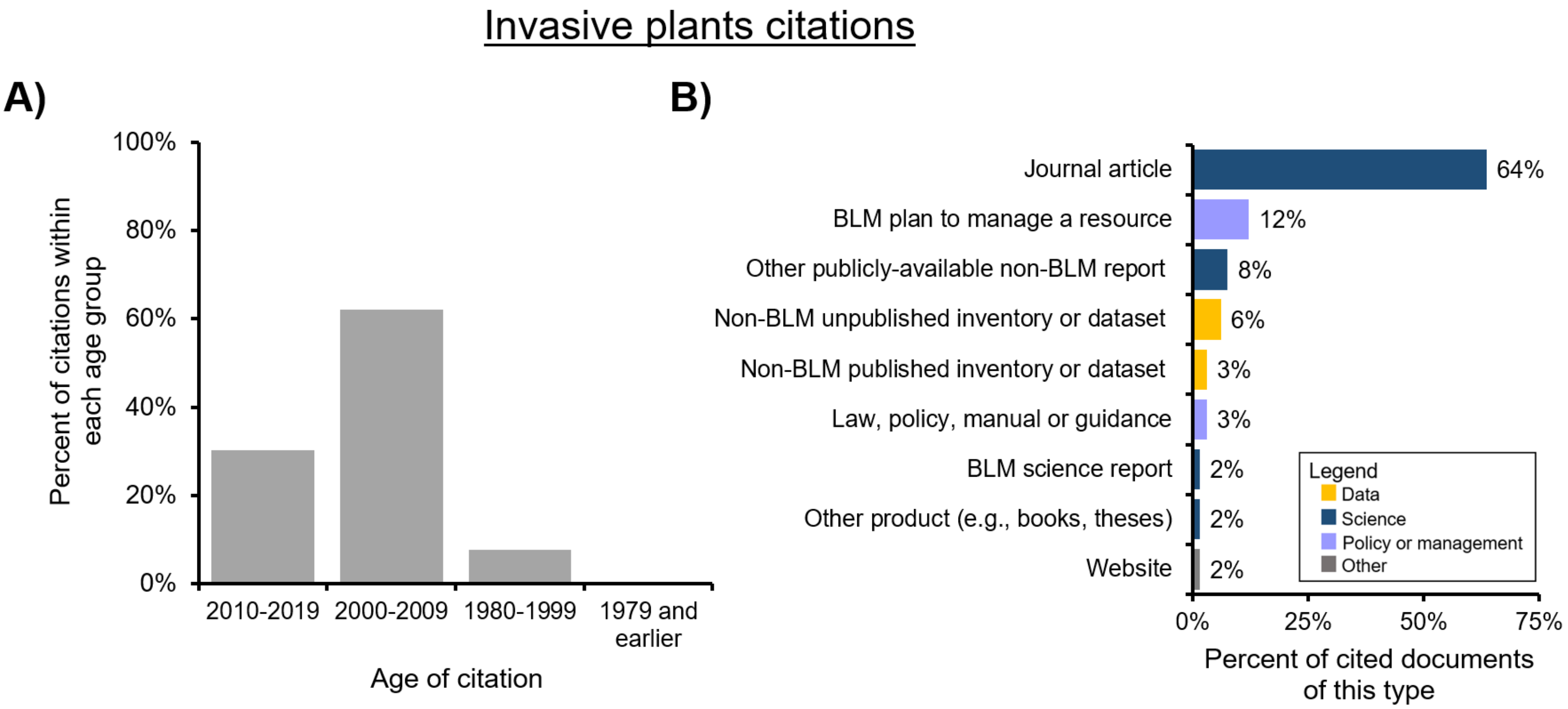


**Figure S7. Invasive plant citations in Bureau of Land Management (BLM) Environmental Assessments.** A) Age of citations and B) types of documents cited in invasive plant resource analysis sections (66 total citations). The documents presented are from a stratified random sample of 70 Environmental Assessments completed by the BLM in Colorado from 2015-2019.

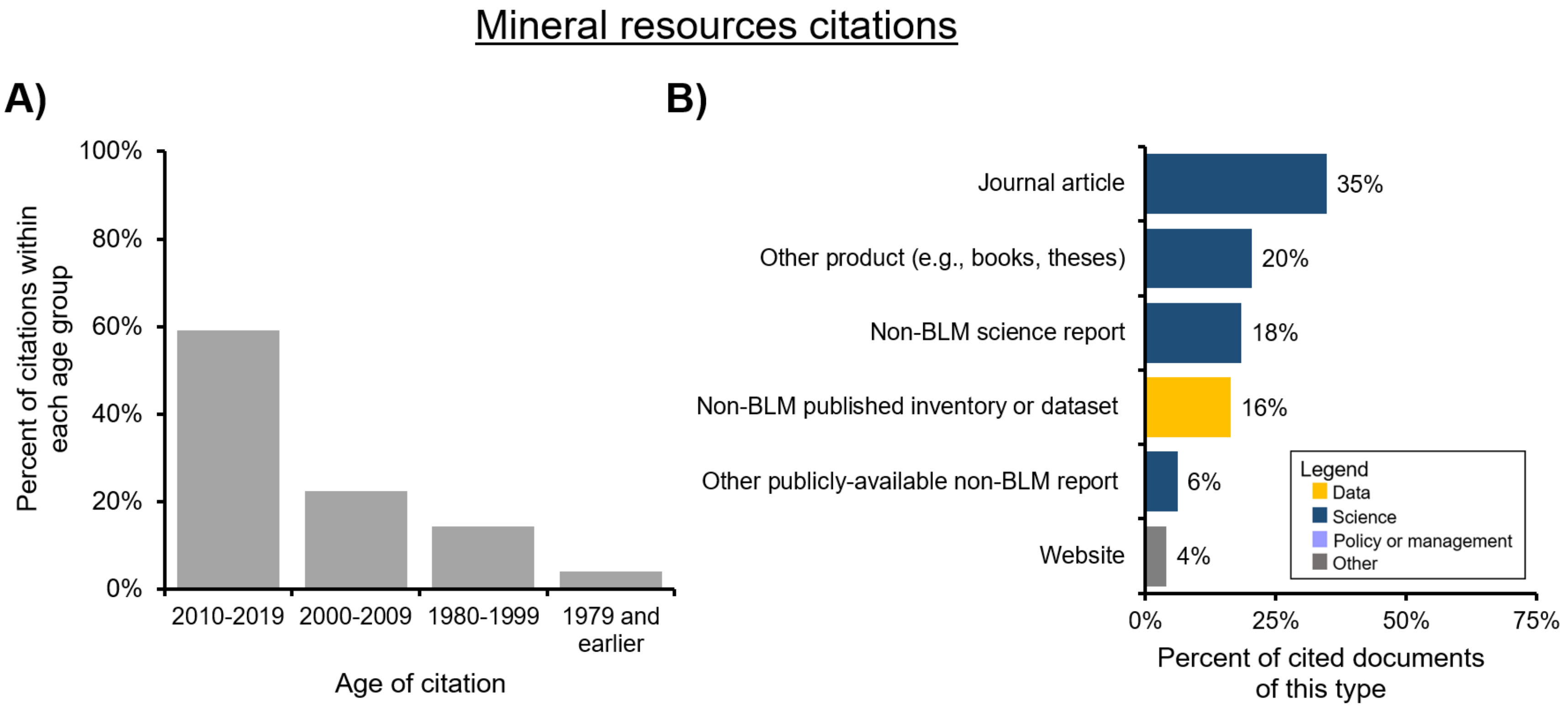


**Figure S8. Mineral resource citations in Bureau of Land Management (BLM) Environmental Assessments.** A) Age of citations and B) types of documents cited in mineral resources analysis sections (49 total citations). The documents presented are from a stratified random sample of 70 Environmental Assessments completed by the BLM in Colorado from 2015-2019.

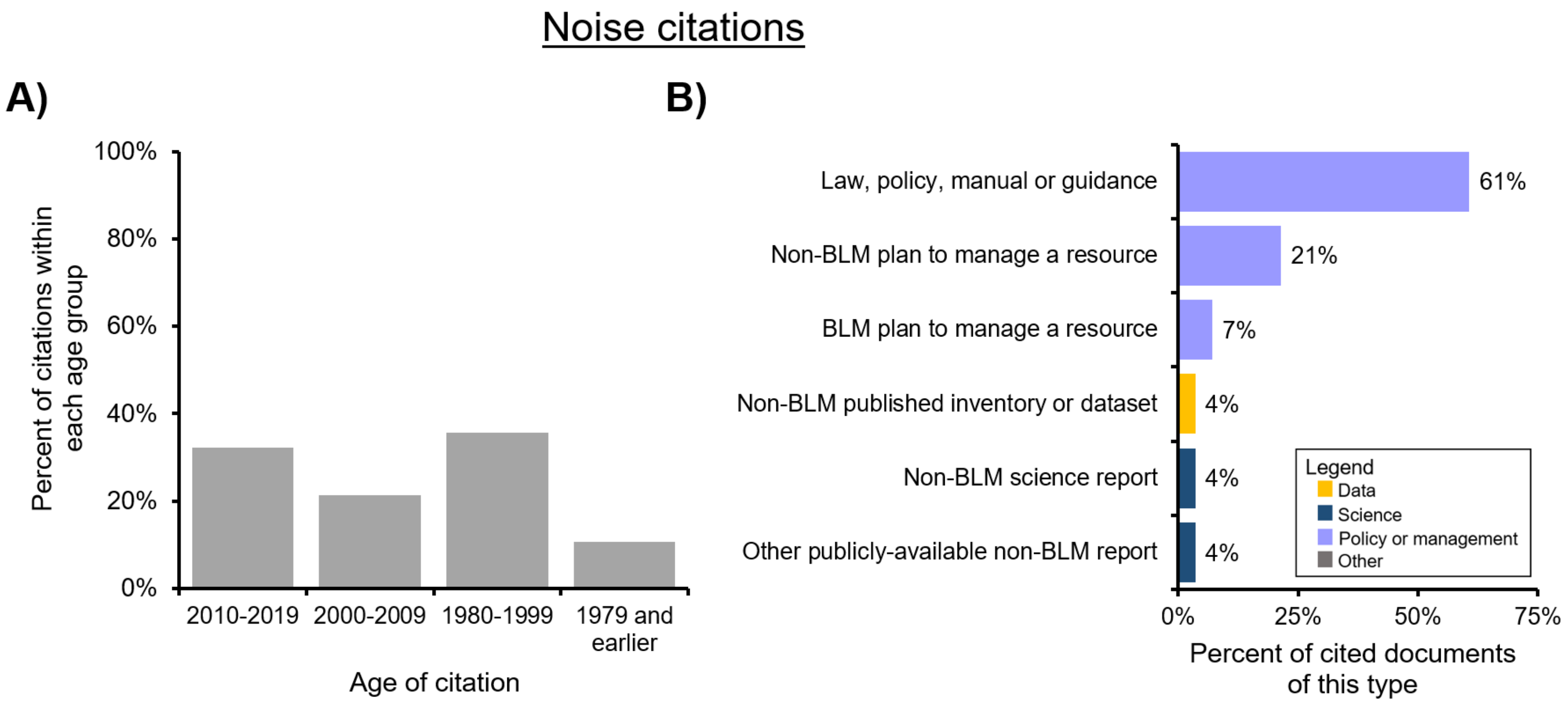


**Figure S9. Noise citations in Bureau of Land Management (BLM) Environmental Assessments.** A) Age of citations and B) types of documents cited in noise resource analysis sections (28 total citations). The documents presented are from a stratified random sample of 70 Environmental Assessments completed by the BLM in Colorado from 2015-2019.

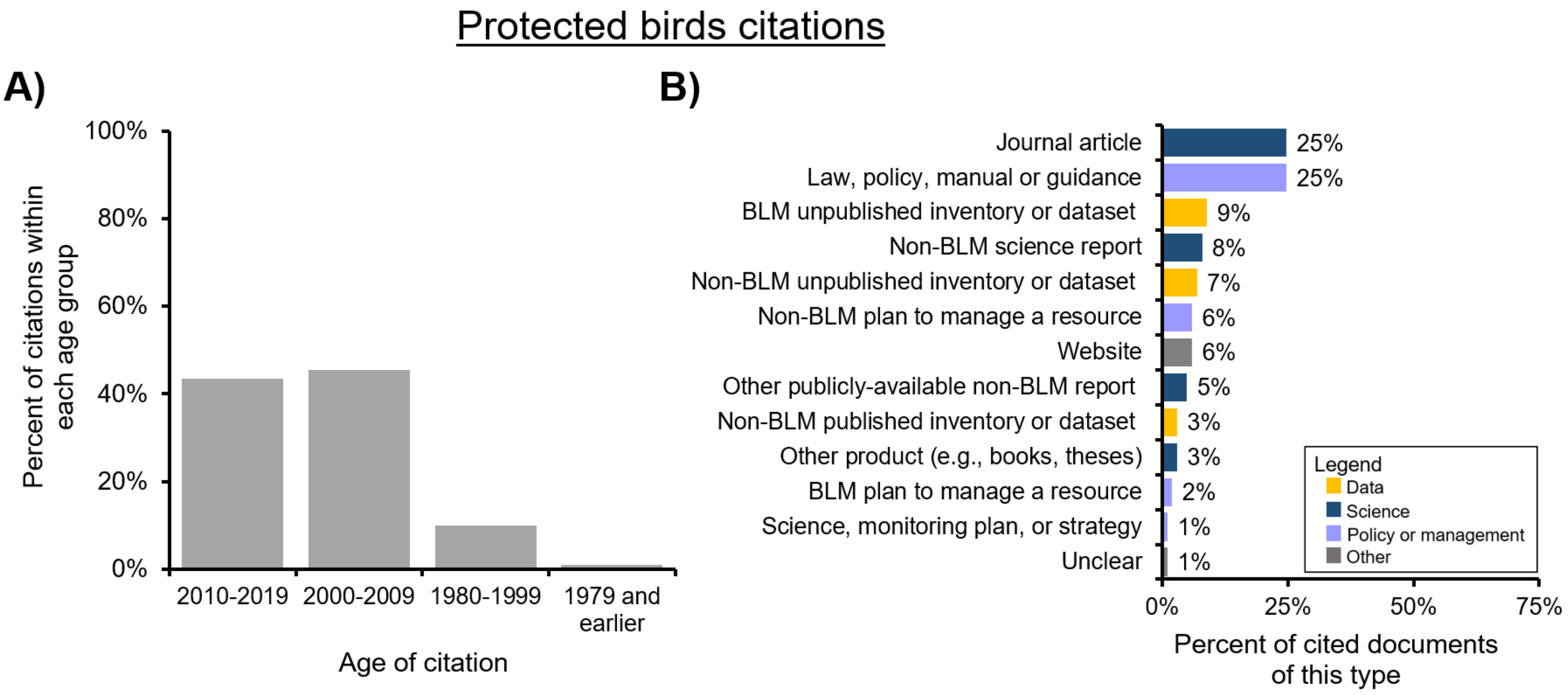


**Figure S10. Protected bird citations in Bureau of Land Management (BLM) Environmental Assessments.** A) Age of citations and B) types of documents cited in protected bird resource analysis sections (101 total citations). The documents presented are from a stratified random sample of 70 Environmental Assessments completed by the BLM in Colorado from 2015-2019.

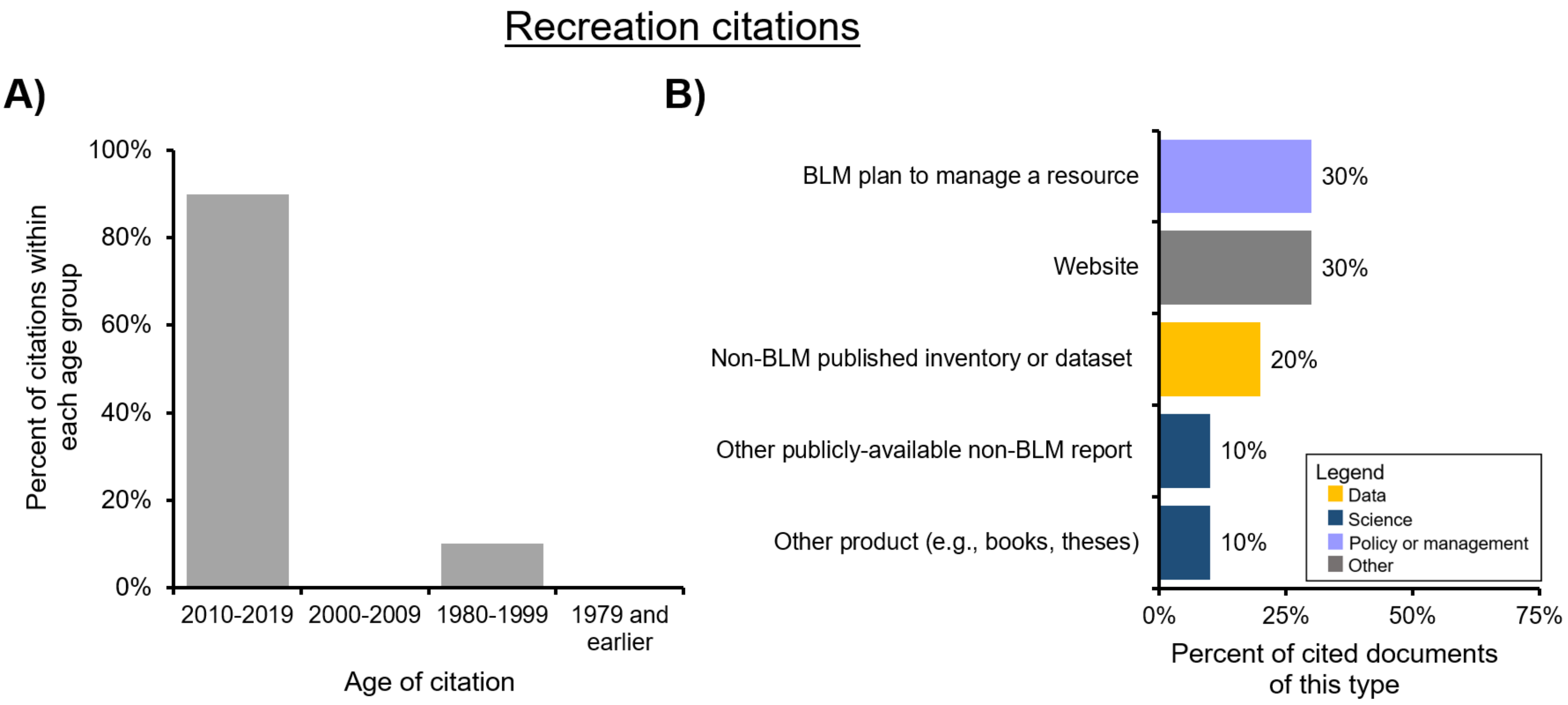


**Figure S11. Recreation citations in Bureau of Land Management (BLM) Environmental Assessments.** A) Age of citations and B) types of documents cited in recreation resource analysis sections (10 total citations). The documents presented are from a stratified random sample of 70 Environmental Assessments completed by the BLM in Colorado from 2015-2019.

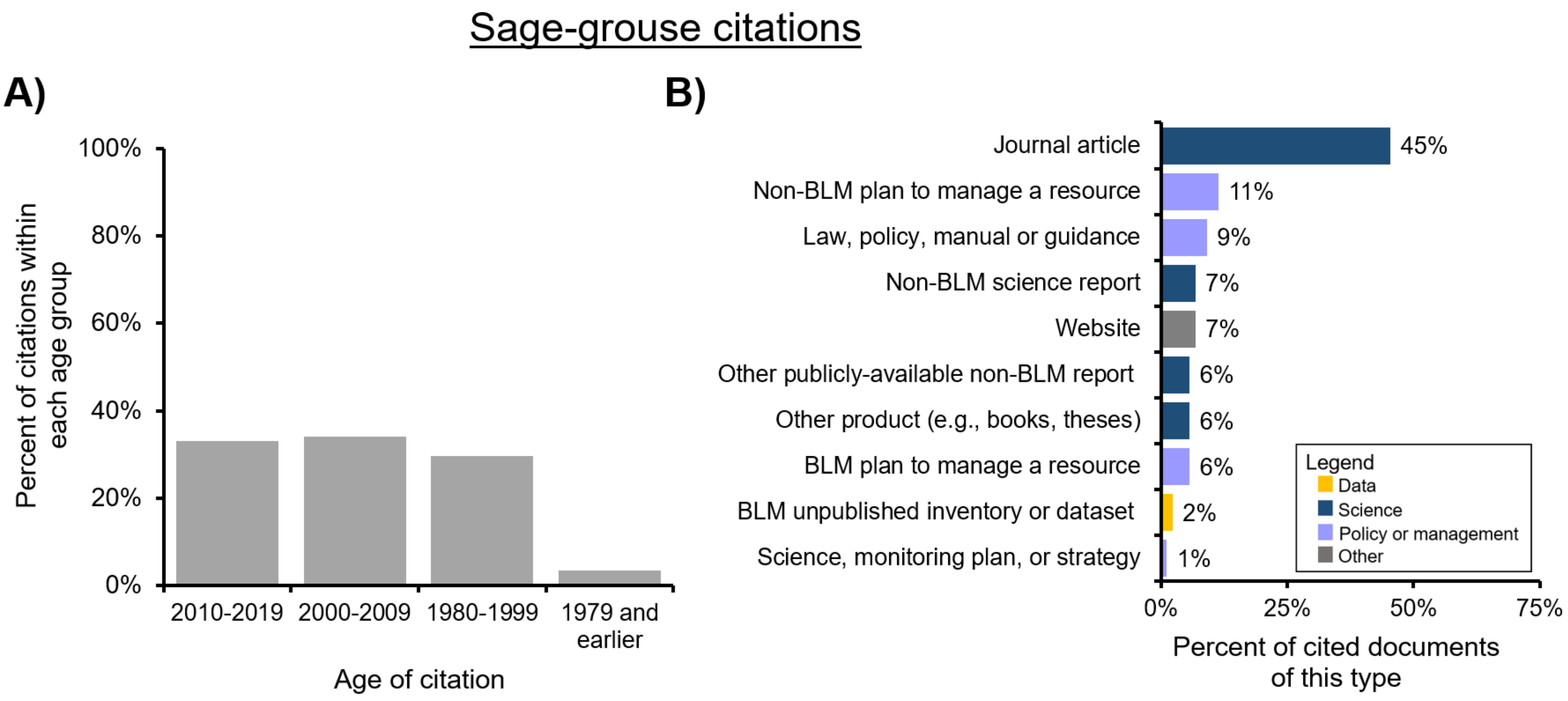


**Figure S12. Sage-grouse citations in Bureau of Land Management (BLM) Environmental Assessments.** A) Age of citations and B) types of documents cited in sage-grouse (*Centrocercus urophasianus* and *Centrocercus minimus*) resource analysis sections (88 total citations). The documents presented are from a stratified random sample of 70 Environmental Assessments completed by the BLM in Colorado from 2015-2019.

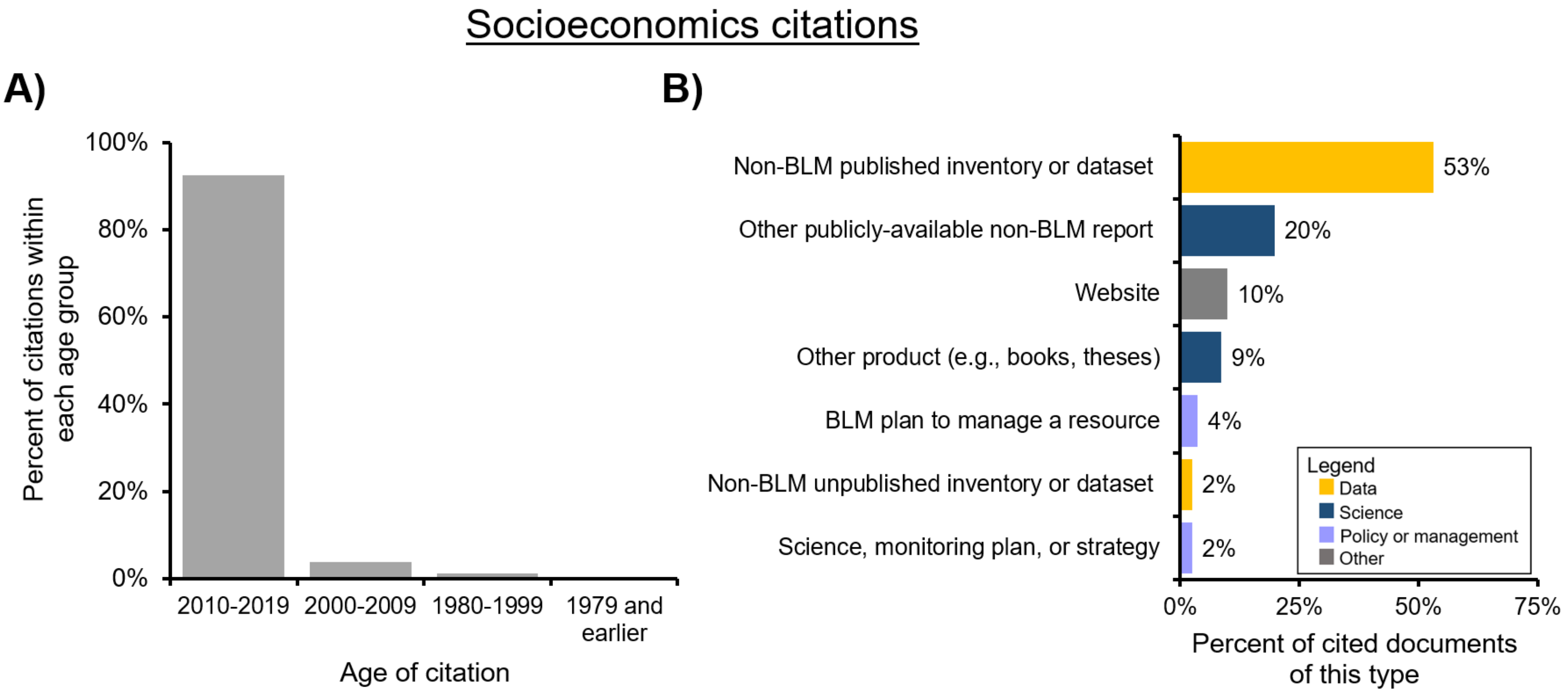


**Figure S13. Socioeconomic citations in Bureau of Land Management (BLM) Environmental Assessments.** A) Age of citations and B) types of documents cited in socioeconomics resource analysis sections (81 total citations). Two citations of unknown age are not included in figure A. The documents presented are from a stratified random sample of 70 Environmental Assessments completed by the BLM in Colorado from 2015-2019.

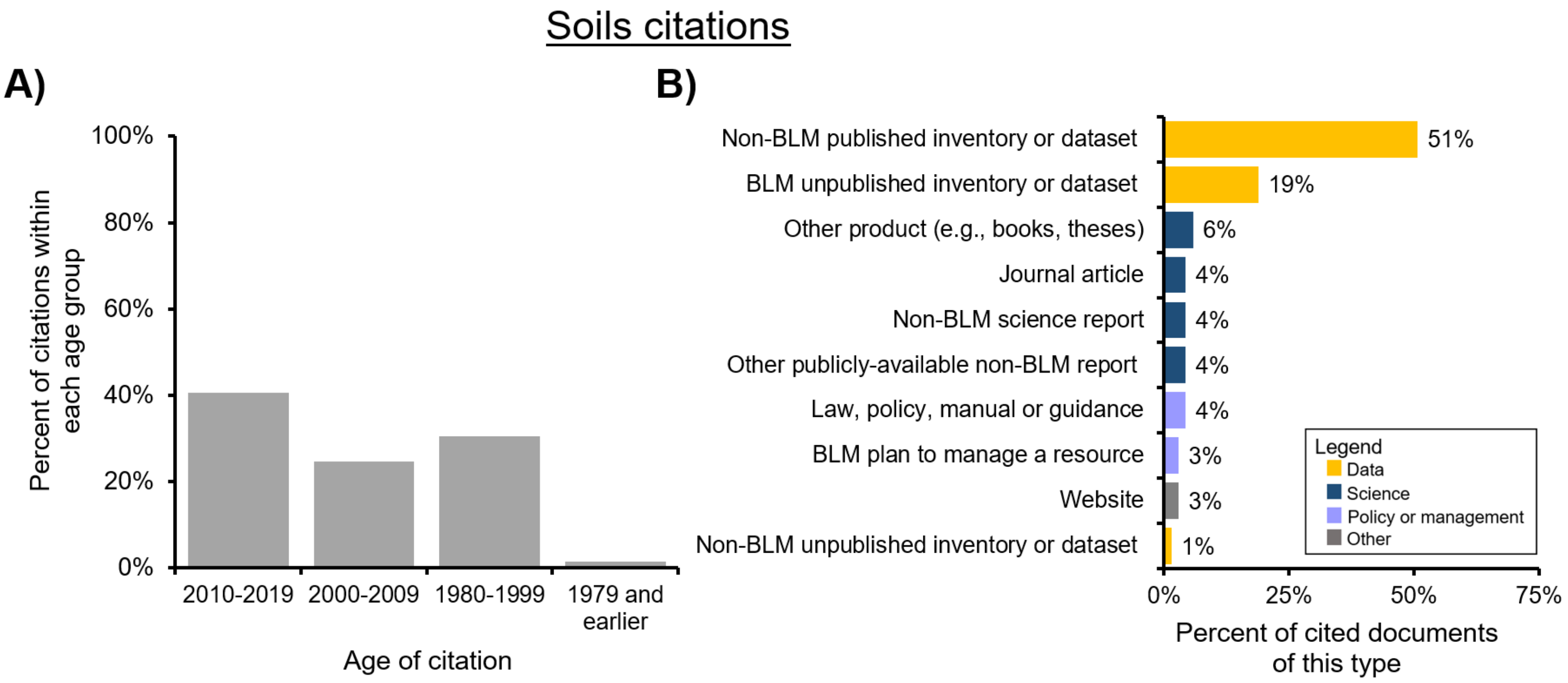


**Figure S14. Soils citations in Bureau of Land Management (BLM) Environmental Assessments.** A) Age of citations and B) types of documents cited in soils resource analysis sections (69 total citations). The documents presented are from a stratified random sample of 70 Environmental Assessments completed by the BLM in Colorado from 2015-2019.

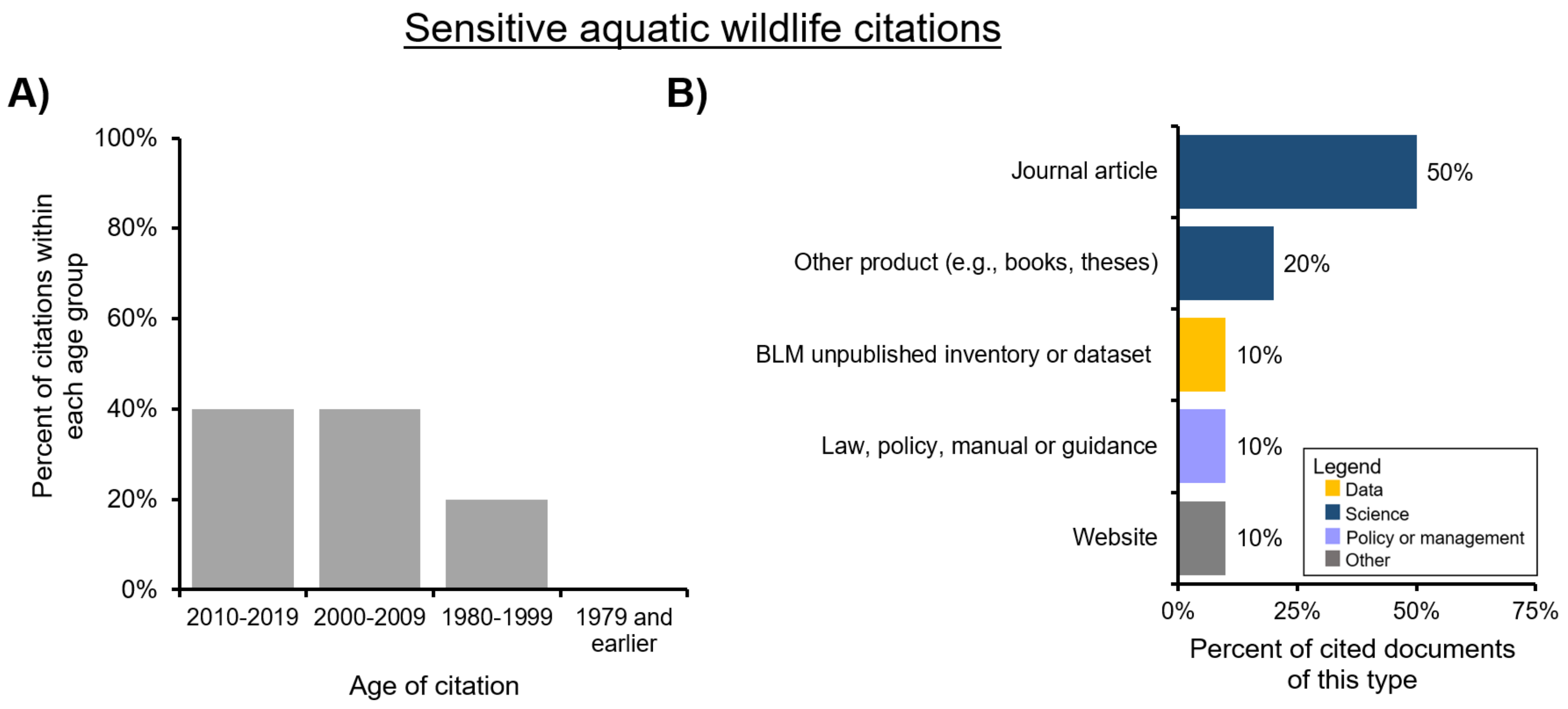


**Figure S15. Sensitive aquatic wildlife citations in Bureau of Land Management (BLM) Environmental Assessments.** A) Age of citations and B) types of documents cited in sensitive aquatic wildlife resource analysis sections (10 total citations). The documents presented are from a stratified random sample of 70 Environmental Assessments completed by the BLM in Colorado from 2015-2019.

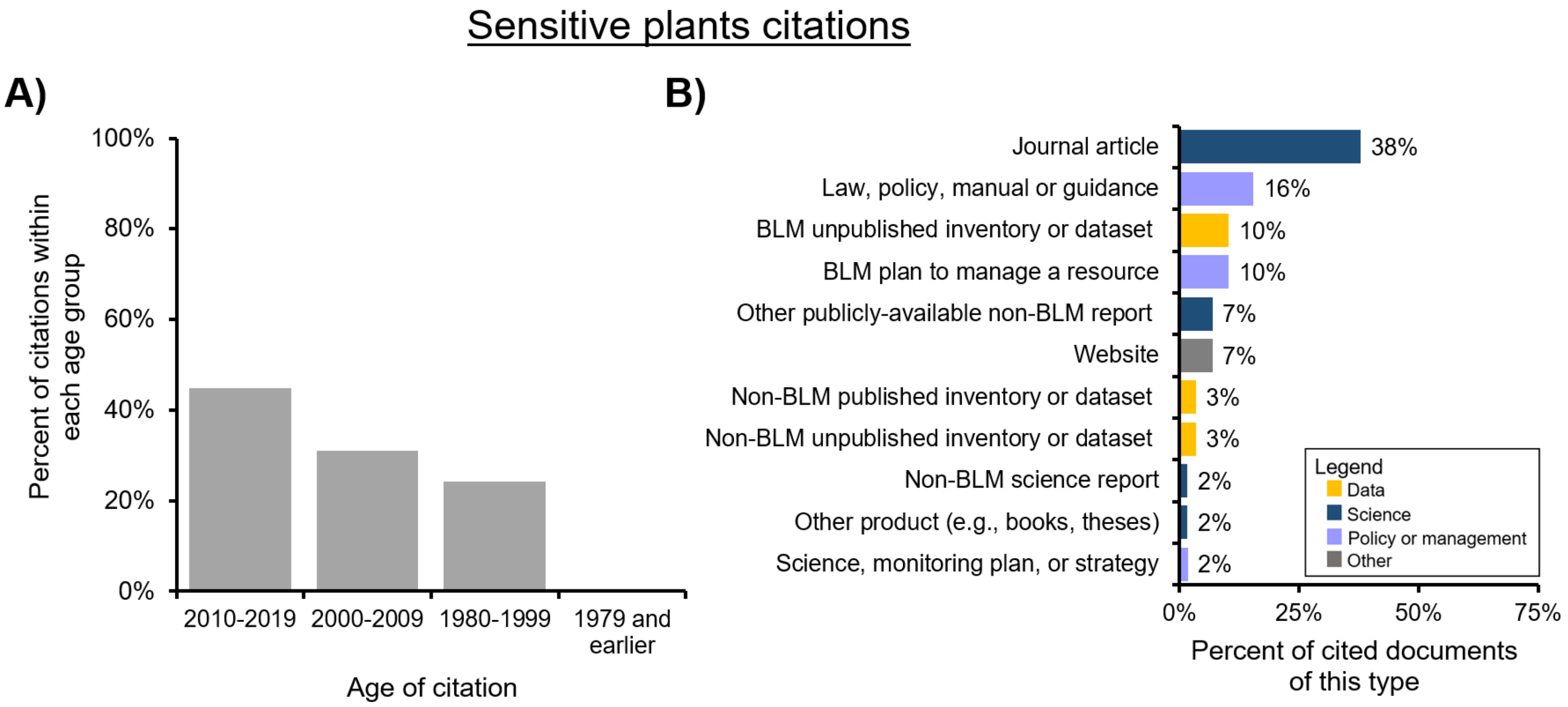


**Figure S16. Sensitive plant citations in Bureau of Land Management (BLM) Environmental Assessments.** A) Age of citations and B) types of documents cited in sensitive plant resource analysis sections (58 total citations). The documents presented are from a stratified random sample of 70 Environmental Assessments completed by the BLM in Colorado from 2015-2019.

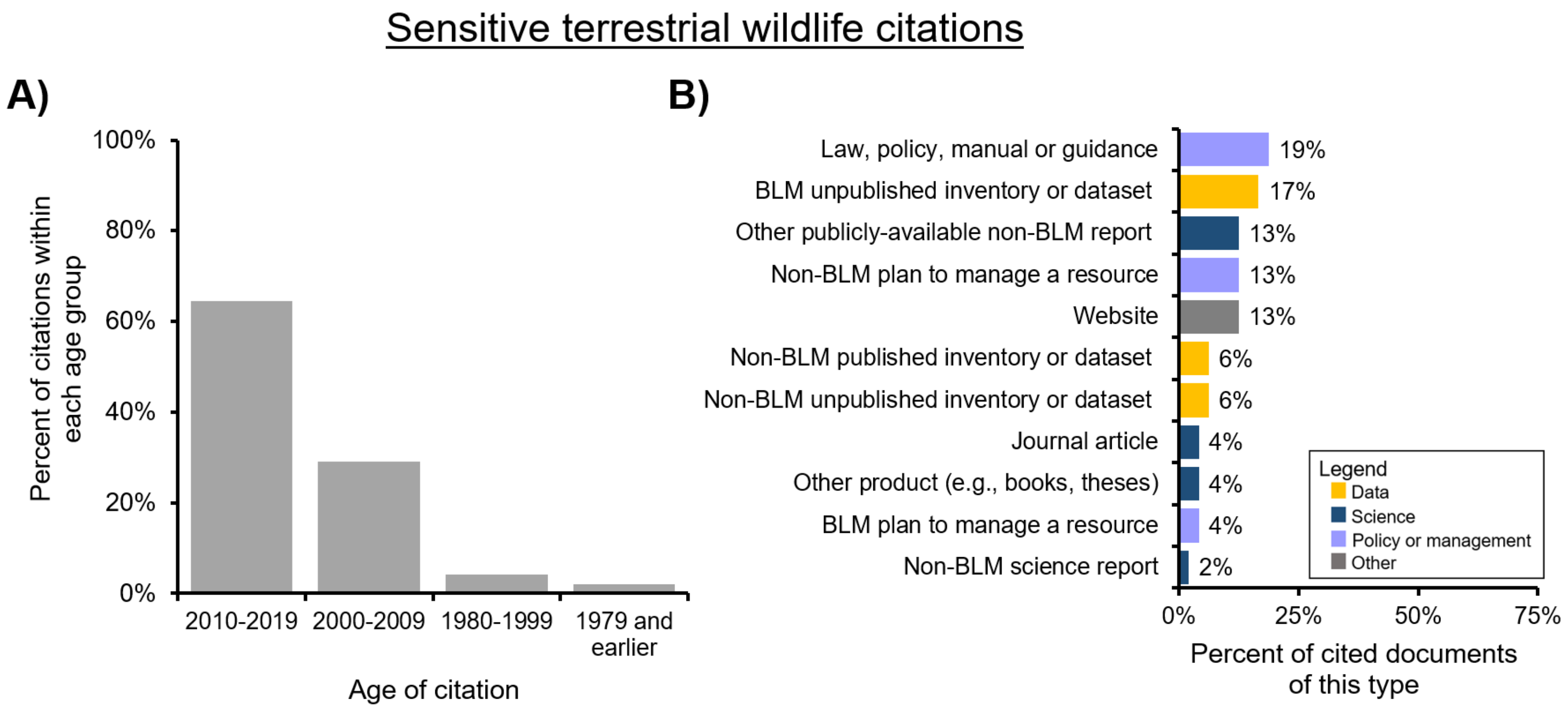


**Figure S17. Sensitive terrestrial wildlife citations in Bureau of Land Management (BLM) Environmental Assessments.** A) Age of citations and B) types of documents cited in sensitive terrestrial wildlife resource analysis sections (48 total citations). The documents presented are from a stratified random sample of 70 Environmental Assessments completed by the BLM in Colorado from 2015-2019.

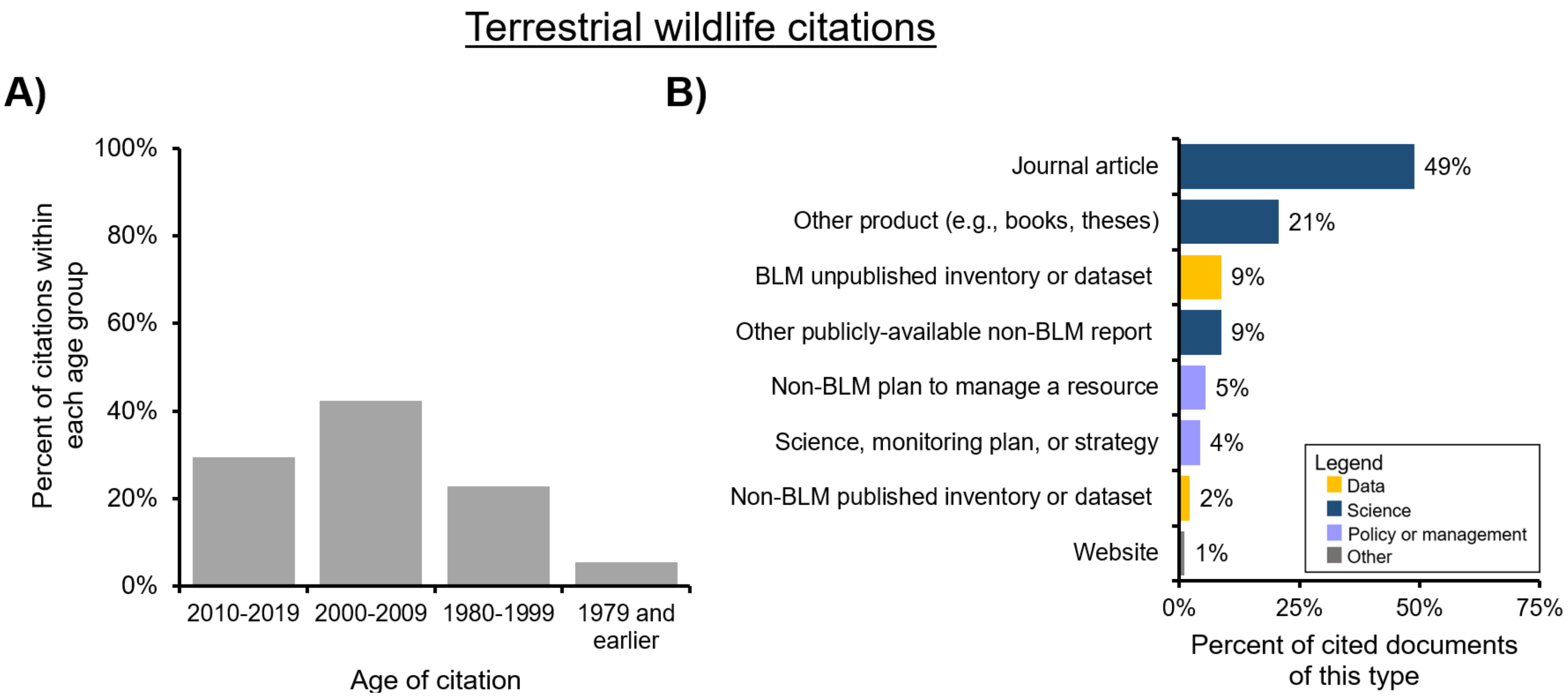


**Figure S18. Terrestrial wildlife citations in Bureau of Land Management (BLM) Environmental Assessments.** A) Age of citations and B) types of documents cited in terrestrial wildlife resource analysis sections (92 total citations). The documents presented are from a stratified random sample of 70 Environmental Assessments completed by the BLM in Colorado from 2015-2019.

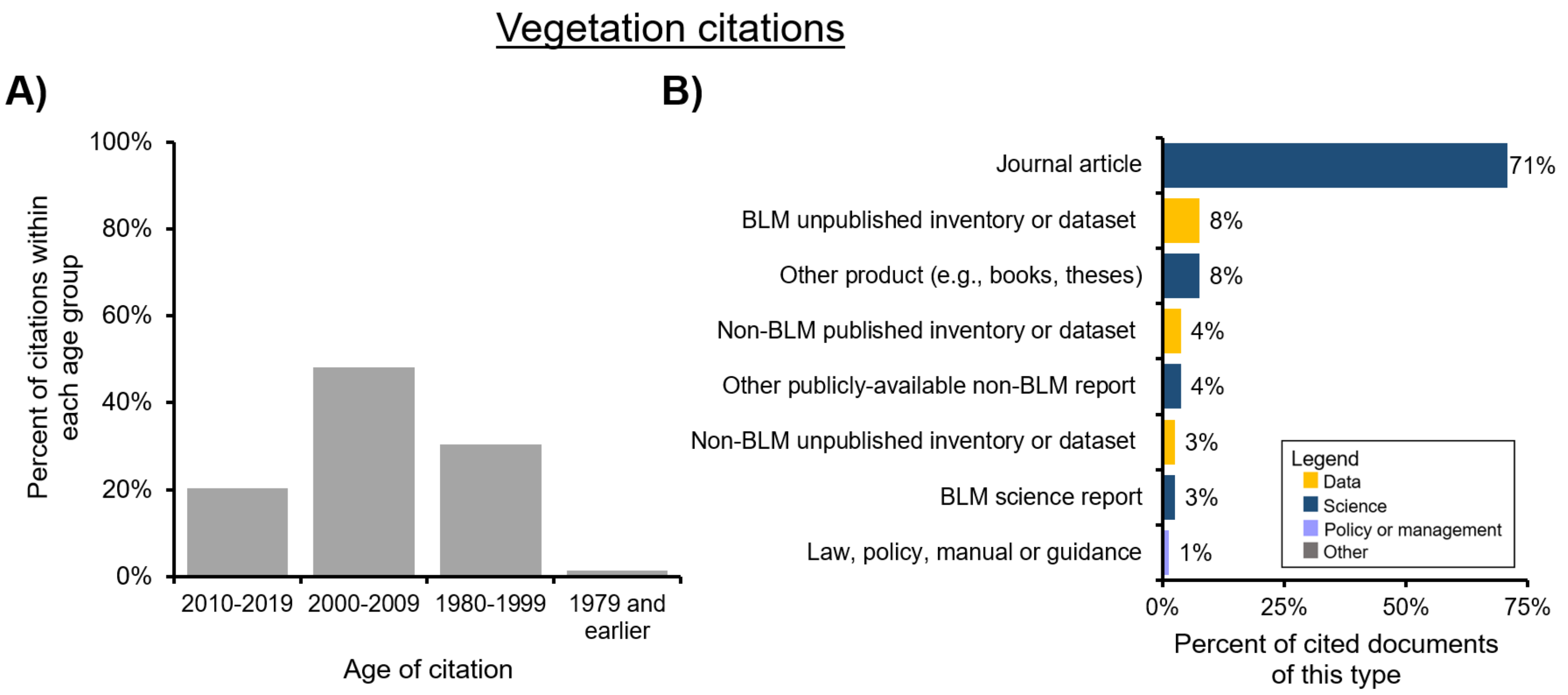


**Figure S19. Vegetation citations in Bureau of Land Management (BLM) Environmental Assessments.** A) Age of citations and B) types of documents cited in vegetation resource analysis sections (79 total citations). The documents presented are from a stratified random sample of 70 Environmental Assessments completed by the BLM in Colorado from 2015-2019.

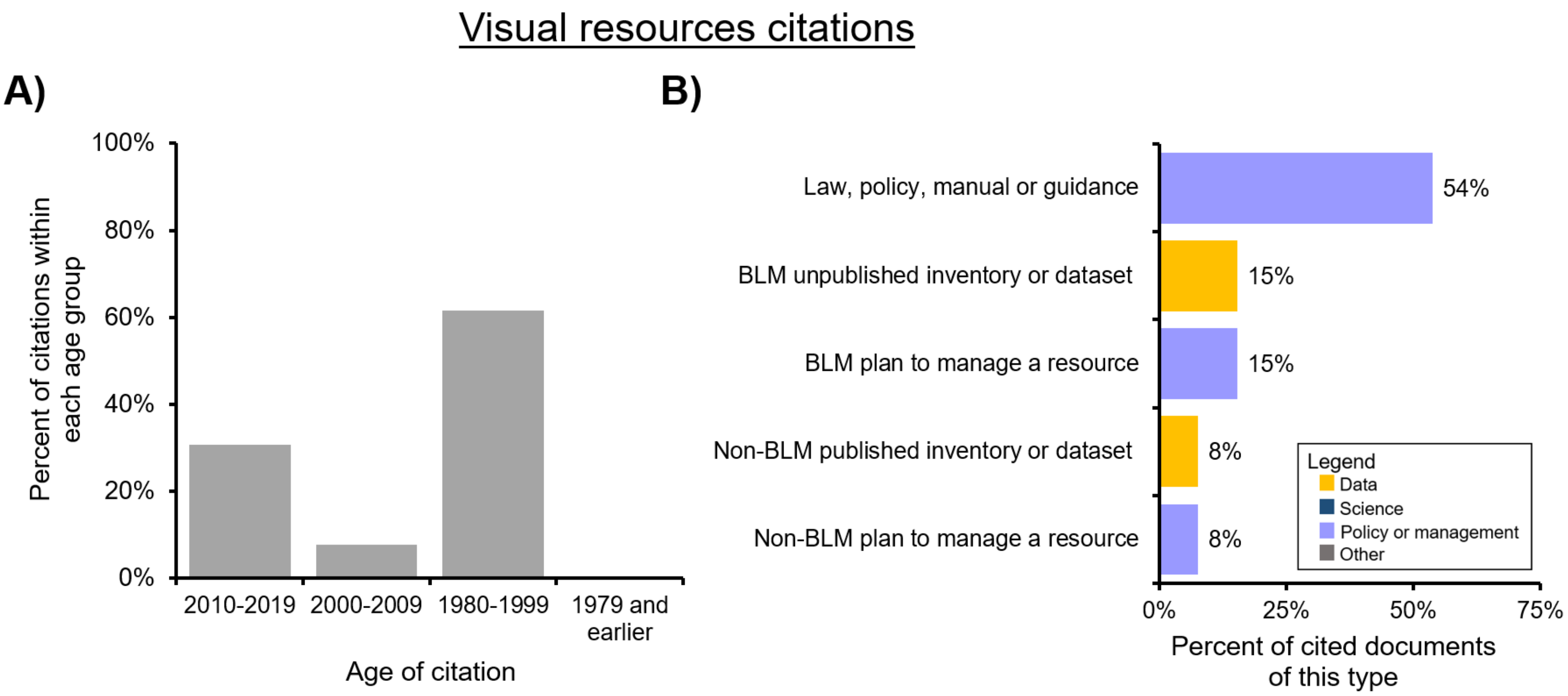


**Figure S20. Visual resources citations in Bureau of Land Management (BLM) Environmental Assessments.** A) Age of citations and B) types of documents cited in visual resources resource analysis sections (13 total citations). The documents presented are from a stratified random sample of 70 Environmental Assessments completed by the BLM in Colorado from 2015-2019.

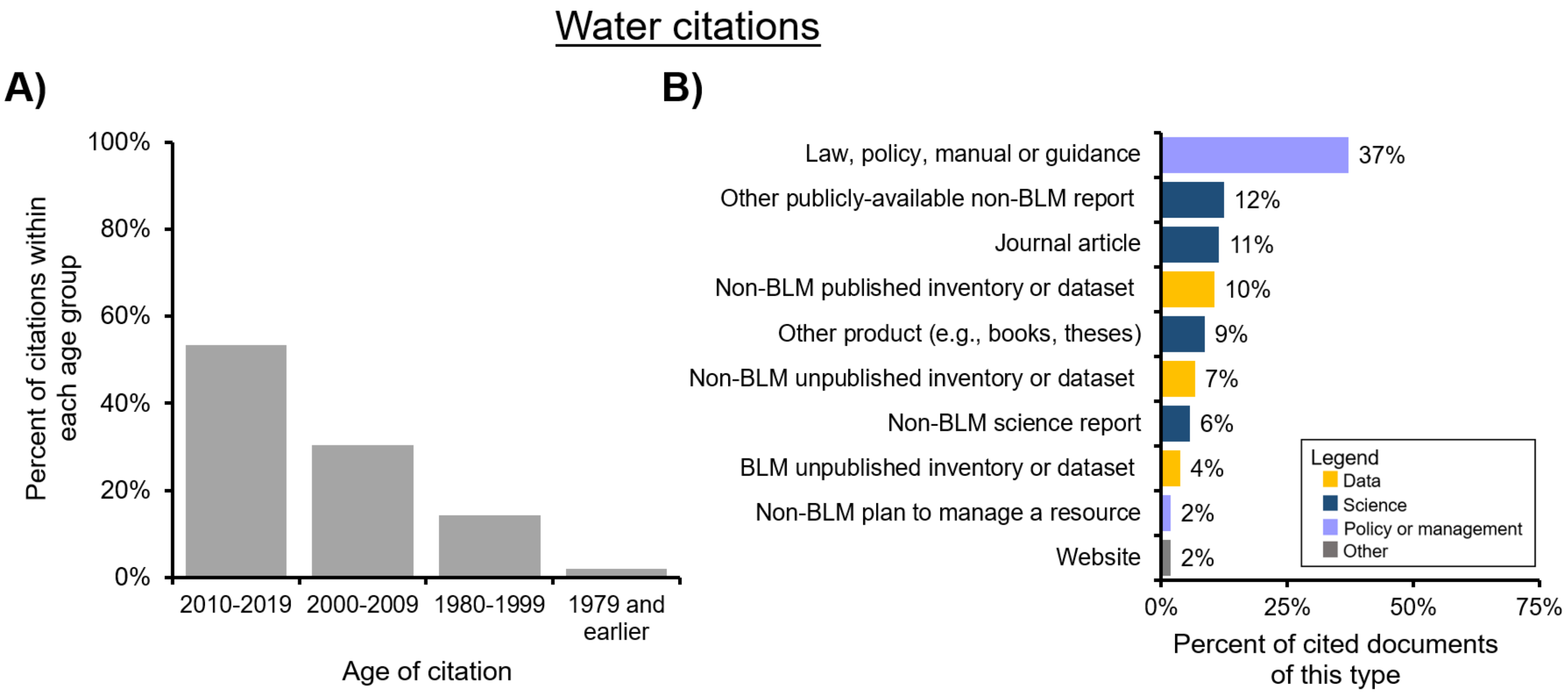


**Figure S21. Water resource citations in Bureau of Land Management (BLM) Environmental Assessments.** A) Age of citations and B) types of documents cited in water resource analysis sections (105 total citations). The documents presented are from a stratified random sample of 70 Environmental Assessments completed by the BLM in Colorado from 2015-2019.

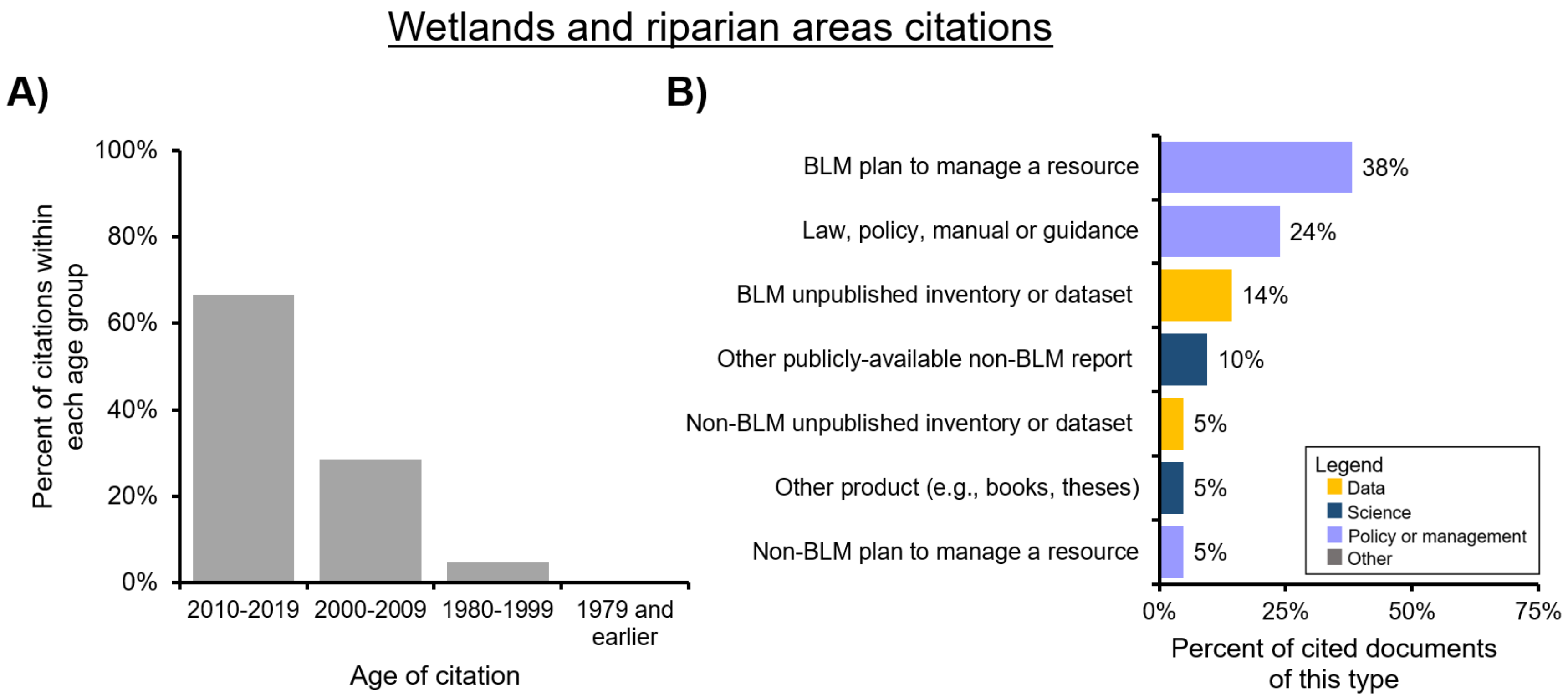


**Figure S22. Wetland and riparian areas citations in Bureau of Land Management (BLM) Environmental Assessments.** A) Age of citations and B) types of documents cited in wetland and riparian areas resource analysis sections (21 total citations). The documents presented are from a stratified random sample of 70 Environmental Assessments completed by the BLM in Colorado from 2015-2019.

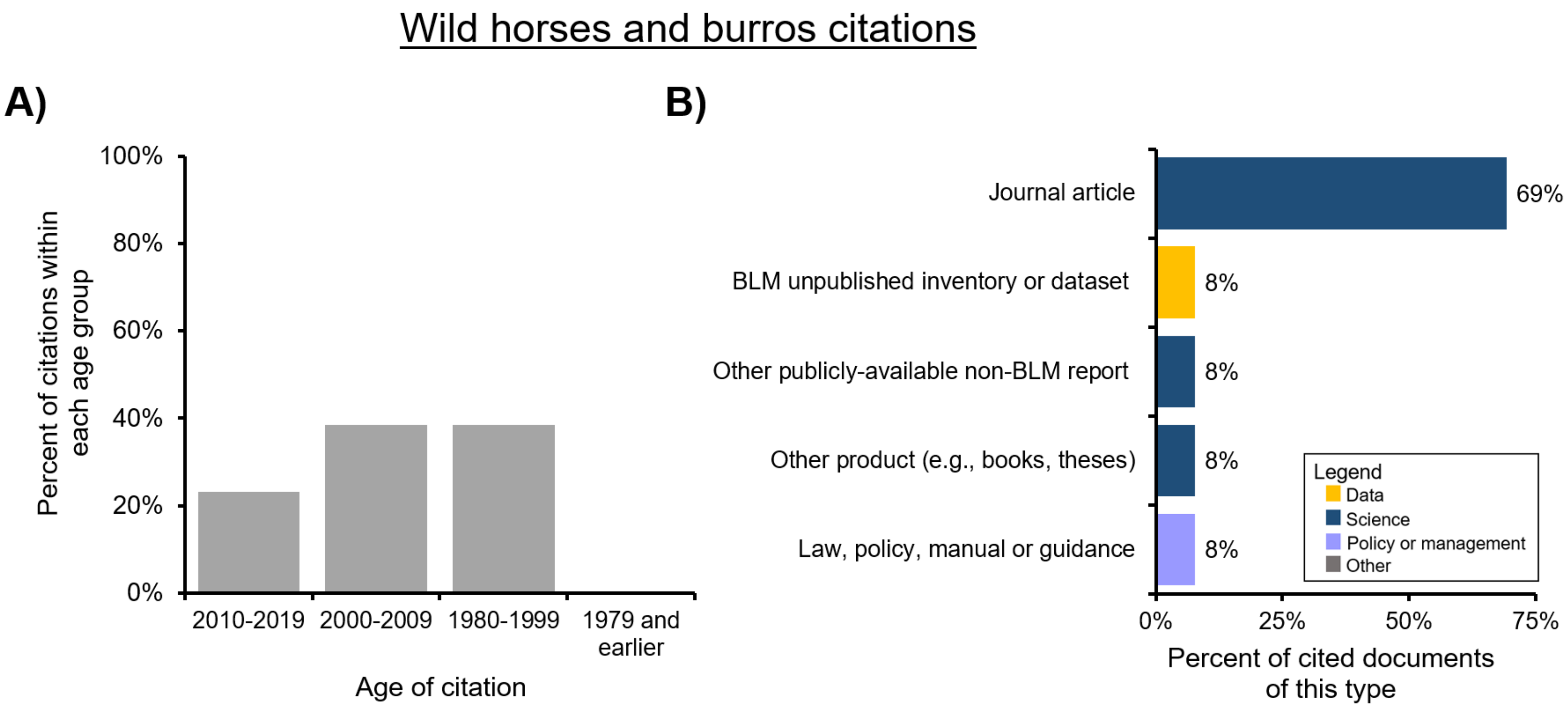


**Figure S23. Wild horse and burro citations in Bureau of Land Management (BLM) Environmental Assessments.** A) Age of citations and B) types of documents cited in wild horse and burro resource analysis sections (13 total citations). The documents presented are from a stratified random sample of 70 Environmental Assessments completed by the BLM in Colorado from 2015-2019.
