# Supplementary material for: Exploring the science and data foundation for Federal public lands decisions
Source: PLoS One. 2025 Feb 10;20(2):e0316013. doi: 10.1371/journal.pone.0316013 (PMC11809896; doi:10.1371/journal.pone.0316013)
Supplement: Attachment — Submitted filename: ResponseToReviewerComments_Fosteretal_PLOSOne_September2024.docx [file pone.0316013.s004.docx]

PONE-D-24-09193

Exploring the science and data foundation for Federal public lands decisions

PLOS ONE

Dear Dr. Foster,

Thank you for submitting your manuscript to PLOS ONE. After careful consideration, we feel that it has merit but does not fully meet PLOS ONE’s publication criteria as it currently stands. Therefore, we invite you to submit a revised version of the manuscript that addresses the points raised during the review process.

I've received sets of comments from two reviewers. Based on their assessments as well as my own reading, I think that your manuscript could be suitable for publication with some relatively modest changes. Foremost, I would like you to consider and address the points raised by Reviewer 1, which -- as was their stated intention -- should further strengthen an already strong manuscript. Overall, your manuscript is well written, so the revisions will mostly involve minor clarifications or additions to your existing material.

Please submit your revised manuscript by Aug 24 2024 11:59PM. If you will need more time than this to complete your revisions, please reply to this message or contact the journal office at [plosone@plos.org](mailto:plosone@plos.org). When you're ready to submit your revision, log on to [https://www.editorialmanager.com/pone/](https://gcc02.safelinks.protection.outlook.com/?url=https%3A%2F%2Fwww.editorialmanager.com%2Fpone%2F&data=05%7C02%7Calison.foster%40usda.gov%7C02aa679c015e40624f5408dca2eb5f35%7Ced5b36e701ee4ebc867ee03cfa0d4697%7C1%7C0%7C638564379573304448%7CUnknown%7CTWFpbGZsb3d8eyJWIjoiMC4wLjAwMDAiLCJQIjoiV2luMzIiLCJBTiI6Ik1haWwiLCJXVCI6Mn0%3D%7C0%7C%7C%7C&sdata=QHNF2ZlY%2BEEi3BpPa9SVeU7JSndpJyGL2jofJkXlCic%3D&reserved=0) and select the 'Submissions Needing Revision' folder to locate your manuscript file.

Please include the following items when submitting your revised manuscript:

- A rebuttal letter that responds to each point raised by the academic editor and reviewer(s). You should upload this letter as a separate file labeled 'Response to Reviewers'.
- A marked-up copy of your manuscript that highlights changes made to the original version. You should upload this as a separate file labeled 'Revised Manuscript with Track Changes'.
- An unmarked version of your revised paper without tracked changes. You should upload this as a separate file labeled 'Manuscript'.

If you would like to make changes to your financial disclosure, please include your updated statement in your cover letter. Guidelines for resubmitting your figure files are available below the reviewer comments at the end of this letter.

If applicable, we recommend that you deposit your laboratory protocols in [protocols.io](https://gcc02.safelinks.protection.outlook.com/?url=http%3A%2F%2Fprotocols.io%2F&data=05%7C02%7Calison.foster%40usda.gov%7C02aa679c015e40624f5408dca2eb5f35%7Ced5b36e701ee4ebc867ee03cfa0d4697%7C1%7C0%7C638564379573315732%7CUnknown%7CTWFpbGZsb3d8eyJWIjoiMC4wLjAwMDAiLCJQIjoiV2luMzIiLCJBTiI6Ik1haWwiLCJXVCI6Mn0%3D%7C0%7C%7C%7C&sdata=0HIuY%2BF6s6cC%2BSrcvPRRjhicrgF7FsHEeqhUHBGdvao%3D&reserved=0) to enhance the reproducibility of your results. Protocols.io assigns your protocol its own identifier (DOI) so that it can be cited independently in the future. For instructions see: [https://journals.plos.org/plosone/s/submission-guidelines#loc-laboratory-protocols](https://gcc02.safelinks.protection.outlook.com/?url=https%3A%2F%2Fjournals.plos.org%2Fplosone%2Fs%2Fsubmission-guidelines%23loc-laboratory-protocols&data=05%7C02%7Calison.foster%40usda.gov%7C02aa679c015e40624f5408dca2eb5f35%7Ced5b36e701ee4ebc867ee03cfa0d4697%7C1%7C0%7C638564379573324187%7CUnknown%7CTWFpbGZsb3d8eyJWIjoiMC4wLjAwMDAiLCJQIjoiV2luMzIiLCJBTiI6Ik1haWwiLCJXVCI6Mn0%3D%7C0%7C%7C%7C&sdata=GRIMuKpSthJUaSDOy8j%2BmN3jJYF01%2BZ8udVLz%2FSs4K4%3D&reserved=0). Additionally, PLOS ONE offers an option for publishing peer-reviewed Lab Protocol articles, which describe protocols hosted on [protocols.io](https://gcc02.safelinks.protection.outlook.com/?url=http%3A%2F%2Fprotocols.io%2F&data=05%7C02%7Calison.foster%40usda.gov%7C02aa679c015e40624f5408dca2eb5f35%7Ced5b36e701ee4ebc867ee03cfa0d4697%7C1%7C0%7C638564379573330051%7CUnknown%7CTWFpbGZsb3d8eyJWIjoiMC4wLjAwMDAiLCJQIjoiV2luMzIiLCJBTiI6Ik1haWwiLCJXVCI6Mn0%3D%7C0%7C%7C%7C&sdata=ewASVn29GTLgxZNgv8z5yy7VRRTk9%2FtvEhw7UjCk%2F0E%3D&reserved=0). Read more information on sharing protocols at [https://plos.org/protocols?utm_medium=editorial-email&utm_source=authorletters&utm_campaign=protocols](https://gcc02.safelinks.protection.outlook.com/?url=https%3A%2F%2Fplos.org%2Fprotocols%3Futm_medium%3Deditorial-email%26utm_source%3Dauthorletters%26utm_campaign%3Dprotocols&data=05%7C02%7Calison.foster%40usda.gov%7C02aa679c015e40624f5408dca2eb5f35%7Ced5b36e701ee4ebc867ee03cfa0d4697%7C1%7C0%7C638564379573335397%7CUnknown%7CTWFpbGZsb3d8eyJWIjoiMC4wLjAwMDAiLCJQIjoiV2luMzIiLCJBTiI6Ik1haWwiLCJXVCI6Mn0%3D%7C0%7C%7C%7C&sdata=QutTwsGQwc3bpyxGrRhIydETcMpYDKzmSU7c%2FCR8dsA%3D&reserved=0).

We look forward to receiving your revised manuscript.

Kind regards,

Frank H. Koch, PhD
Academic Editor
PLOS ONE

Journal Requirements:

When submitting your revision, we need you to address these additional requirements.

1. Please ensure that your manuscript meets PLOS ONE's style requirements, including those for file naming. The PLOS ONE style templates can be found at 
[https://journals.plos.org/plosone/s/file?id=wjVg/PLOSOne_formatting_sample_main_body.pdf](https://gcc02.safelinks.protection.outlook.com/?url=https%3A%2F%2Fjournals.plos.org%2Fplosone%2Fs%2Ffile%3Fid%3DwjVg%2FPLOSOne_formatting_sample_main_body.pdf&data=05%7C02%7Calison.foster%40usda.gov%7C02aa679c015e40624f5408dca2eb5f35%7Ced5b36e701ee4ebc867ee03cfa0d4697%7C1%7C0%7C638564379573340879%7CUnknown%7CTWFpbGZsb3d8eyJWIjoiMC4wLjAwMDAiLCJQIjoiV2luMzIiLCJBTiI6Ik1haWwiLCJXVCI6Mn0%3D%7C0%7C%7C%7C&sdata=pFRkd60LXqusqsUwxW9ehToYiTP9TwYRalPEcqU5vFs%3D&reserved=0) and 
[https://journals.plos.org/plosone/s/file?id=ba62/PLOSOne_formatting_sample_title_authors_affiliations.pdf](https://gcc02.safelinks.protection.outlook.com/?url=https%3A%2F%2Fjournals.plos.org%2Fplosone%2Fs%2Ffile%3Fid%3Dba62%2FPLOSOne_formatting_sample_title_authors_affiliations.pdf&data=05%7C02%7Calison.foster%40usda.gov%7C02aa679c015e40624f5408dca2eb5f35%7Ced5b36e701ee4ebc867ee03cfa0d4697%7C1%7C0%7C638564379573346361%7CUnknown%7CTWFpbGZsb3d8eyJWIjoiMC4wLjAwMDAiLCJQIjoiV2luMzIiLCJBTiI6Ik1haWwiLCJXVCI6Mn0%3D%7C0%7C%7C%7C&sdata=2F5%2BIJXbG0m1Srfxja6jI24Nxr4RNvY7YGAvnqHRuAQ%3D&reserved=0)

2. When completing the data availability statement of the submission form, you indicated that you will make your data available on acceptance. We strongly recommend all authors decide on a data sharing plan before acceptance, as the process can be lengthy and hold up publication timelines. Please note that, though access restrictions are acceptable now, your entire data will need to be made freely accessible if your manuscript is accepted for publication. This policy applies to all data except where public deposition would breach compliance with the protocol approved by your research ethics board. If you are unable to adhere to our open data policy, please kindly revise your statement to explain your reasoning and we will seek the editor's input on an exemption. Please be assured that, once you have provided your new statement, the assessment of your exemption will not hold up the peer review process.

**Additional Editor Comments:**

Specific comments:
Line 77 - delete comma after "Independent" (I don't think that it's necessary)

*Response*: Comma deleted
Table 1 - in the first two "Description of document type" boxes, change "that is" to "that are"

*Response*: Change made
Line 402 - "socioeconomic" instead of "socioeconomics"

*Response:* Change made
In the supplemental information, I suggest adding a title for each S1-S23 Fig., so readers don't have to flip back to the master list on p.5.

*Response:* We agree that this would be helpful for readers and have added a title to each figure.


[Note: HTML markup is below. Please do not edit.]

Reviewers' comments:

Reviewer's Responses to Questions

**Comments to the Author**

1. Is the manuscript technically sound, and do the data support the conclusions?

The manuscript must describe a technically sound piece of scientific research with data that supports the conclusions. Experiments must have been conducted rigorously, with appropriate controls, replication, and sample sizes. The conclusions must be drawn appropriately based on the data presented.

Reviewer #1: Yes

Reviewer #2: Yes

2. Has the statistical analysis been performed appropriately and rigorously?

Reviewer #1: I Don't Know

Reviewer #2: Yes

3. Have the authors made all data underlying the findings in their manuscript fully available?

The [PLOS Data policy](https://gcc02.safelinks.protection.outlook.com/?url=http%3A%2F%2Fwww.plosone.org%2Fstatic%2Fpolicies.action%23sharing&data=05%7C02%7Calison.foster%40usda.gov%7C02aa679c015e40624f5408dca2eb5f35%7Ced5b36e701ee4ebc867ee03cfa0d4697%7C1%7C0%7C638564379573351825%7CUnknown%7CTWFpbGZsb3d8eyJWIjoiMC4wLjAwMDAiLCJQIjoiV2luMzIiLCJBTiI6Ik1haWwiLCJXVCI6Mn0%3D%7C0%7C%7C%7C&sdata=UbBEcfIGqkAvoj9ZvVpcLln0eZYR1eYeB5%2BMAbXwDl4%3D&reserved=0) requires authors to make all data underlying the findings described in their manuscript fully available without restriction, with rare exception (please refer to the Data Availability Statement in the manuscript PDF file). The data should be provided as part of the manuscript or its supporting information, or deposited to a public repository. For example, in addition to summary statistics, the data points behind means, medians and variance measures should be available. If there are restrictions on publicly sharing data—e.g. participant privacy or use of data from a third party—those must be specified.

Reviewer #1: Yes

Reviewer #2: Yes

4. Is the manuscript presented in an intelligible fashion and written in standard English?

PLOS ONE does not copyedit accepted manuscripts, so the language in submitted articles must be clear, correct, and unambiguous. Any typographical or grammatical errors should be corrected at revision, so please note any specific errors here.

Reviewer #1: Yes

Reviewer #2: Yes

5. Review Comments to the Author

Please use the space provided to explain your answers to the questions above. You may also include additional comments for the author, including concerns about dual publication, research ethics, or publication ethics. (Please upload your review as an attachment if it exceeds 20,000 characters)

Reviewer #1: Thank you for the opportunity to review this manuscript. It is a well-written and clear contribution the emerging literature on the use of science in agency decision-making. The authors gather and code citations from 70 Environmental Assessments (EAs) from the BLM in Colorado from 2015-2019. They analyze the age, type, and frequency of citations across (a) proposed actions and (b) resources that could be affected by the proposed actions. The manuscript provides a rich scholarly lens in which to contextualize new descriptive research findings. Overall, the manuscript is strong. However, I have a few comments and questions that I hope help to improve the contribution:

Response to reviewer #1: Thank you for the favorable review and constructive feedback. We really appreciate the time that reviewer 1 clearly took in reviewing the manuscript and feel that the paper is much improved as a result of their feedback. Thank you, reviewer 1 for your thoughtful insight! When reading our responses, please note that line numbers refer to the version of the manuscript with track changes.

1) Given that the “introduction” section covers a lot of theoretical material and prior research (and is less of an outline), I suggest titling it according to the content or puzzle the authors address in the manuscript. Alternatively, they could perhaps include a very brief introduction section and save the bulk of the literature for a section on that topic.

*Response*: We agree with the reviewer that the introduction section has a large focus on existing research and that much of the focus is to set the stage for the study rather than outlining the study itself. However, we feel that this approach is appropriate since we take an unusual approach and use an atypical data set, meaning that there is a greater need to contextualize the data (Environmental Assessments) than there may be in a typical paper. Additionally, this approach is in keeping with PLOSOne’s guidelines, which state that the introduction should “provide background that puts in the manuscript in context, define the problem addressed and why it is important, include a brief review of key literature, note any relevant controversies or disagreements in the field, conclude with a brief statement of the overall aim of the work.” We feel that the introduction meets these objectives and have not restructured the introduction.

2) Potentially one of the more important comments among my comments/questions: In the introductory paragraph starting on line 71, the authors define what they (and the law) mean by “best available science”. While they acknowledge that “different groups can have different perceptions of what is most important in characterizing best available science”, the authors do not dive any deeper into the literature on the bias toward Western science and ways of knowing. Given the recent discussions by federal agencies, universities, and researchers on the bias toward Western knowledge production and use in policy and management, the authors should spend a paragraph or two addressing this issue. This seems especially important given the authors’ later focus on the lack of Indigenous perspectives.

*Response:* Thank you for this comment. The reviewer raises a great point, and we agree that we should address indigenous knowledge and ways of knowing. To address this issue, we have added discussion of indigenous knowledge to the introduction and discussion. In the introduction (lines 64-68 and copied below) we added a sentence citing recent U.S. presidential administration requirements to consider indigenous knowledge in federal decision-making and that the importance of indigenous knowledge has recently gained more attention.

“The importance and value of traditional indigenous knowledge has recently gained attention within the scientific community (10, 11) and the Biden administration has released numerous executive orders, memos, and guidance documents recognizing the importance of indigenous knowledge and instructed agencies to consider it in their decision-making (12-15).”

In the discussion (lines 498-506 and copied below) we added a paragraph discussing the importance of indigenous knowledge and our failure to specifically address it in our study.

“We were also limited in our ability to speak to citations of indigenous knowledge. Our decision-tree was primarily framed around document types used in western science and did not have specific categories for indigenous knowledge or ways of knowing. The U.S. federal government’s recent focus on indigenous knowledge pre-dated our study. Indigenous knowledge should be considered and included in resource analyses regardless of federal regulation and policy, however the information incorporated in analyses is often driven by federal requirements. We think that the incorporation and use of indigenous knowledge in environmental analyses should be a topic addressed in future study, and encourage potential users of our decision tree to consider adding a category for indigenous knowledge.”

3) The authors justify the study case, in part because of their established relationships with BLM staff. Can the authors address what may have been lost had the study not engaged a co-production model? The approach certainly seems to provide benefits in terms of the ability to build capital and share findings with decision-makers, but the authors should address what was gained through partnership (perhaps things like better understanding citation types).

*Response*: The reviewer asks a good question and we agree that it is worthwhile to address what may have been lost if we had not engaged in coproduction. We added text to the discussion that describes these benefits (lines 507-520 and copied below).

“Coproduction was invaluable to all aspects of our study, from methods development to interpretation of results. Working in close alignment with BLM staff ensured that our findings were structured and categorized in way that was interpretable and usable by BLM staff. We may not have collected data on both actions and resources had we not worked in coproduction with BLM, yet understanding citations for each of these categories was an important distinction in the context of NEPA. Coproduction also aided in understanding the action and resource categories used in this study, as it was difficult to understand some of the categories without the context provided by BLM staff and this context was essential to consistent coding of citations. Also, the relationships built through coproduction allowed for open communication on how we may share practical information about science use in decision-making that was not perceived as threatening or judgmental. All parties, and USGS scientists especially, gained a highly improved understanding of the NEPA process and how science is likely to be used and documented by staff. This learning has led to a fundamental change in how USGS staff communicate and share findings with resource managers.”

4) The authors focus on EAs. Besides EAs being more common, why not EISs as well? Do the authors expect any differences between the two types of analyses?

*Response:* We focused on EAs as EISs are substantially different documents. By definition in the NEPA, EISs are used for projects that are anticipated to have significant environmental impacts where EAs are used where no significant impacts are anticipated or the degree of impact is uncertain. Most proposed actions are analyzed via EAs and EAs are the environmental analysis document used by BLM field staff most commonly and thus most reflective of BLM use of science and data. When EISs are needed, it's common for these documents to be completed by contractors, meaning that BLM staff are often not the primary authors. Given that interest in this project was expressed by BLM staff we wanted to focus on the analyses more frequently completed by BLM staff.

We do anticipate that our results would have differed if we had looked at EISs instead of EAs. As stated previously, EAs and EISs have different purposes (to determine if there will be a significant effect vs. what the significant effect will be), which lends itself toward differing analyses. Additionally, we expect that results would differ given that EISs are often completed by contractors versus EAs which are typically written by BLM staff. The science and data available to BLM staff and contractors is likely different – some BLM data may only be available internally or a contractor may have greater access to science information that is behind a pay wall – and we expect that this would be reflected in EAs and EISs.

5) The authors focus on citations within only the “main body” of the EA, excluding information from the “purpose and need” and other sections as well as from Appendices.
a. First, I think it would be very interesting to know what citations come up in the “purpose and need” section because this section frames the problem – and could therefore be particularly important. However, I understand this may be outside the scope of the current analysis.

b. Second, my hunch is that a lot of citations are included in the Appendices. The authors could take a sub-sample of their documents and examine the extent that Appendices include citations to address this issue. If not that, are there reasons to be concerned that the science is more recent, a different type, etc. in the Appendices? Or that citations look different across resources or action purpose in Appendices?

*Response:* Yes, it is possible that citations are used within the purpose and need section of the EA, as well as in the appendices. The coproduction team decided early on to only assess citations found within the main body of the analysis sections. We made some tradeoffs in developing our methods and by limiting ourselves to analysis sections we were able to increase our sample size and sample a wide range of action categories. Further, this approach is what was advocated by BLM staff - that we should focus on resource analyses sections and sample all commonly proposed action categories.

As to looking at citations in the purpose and need, it would have been difficult to summarize and interpret citations from this section. The purpose and need focuses on the proposed action, which is often narrowly focused on a single activity, ecosystem type, or location. Additionally, there are typically substantially fewer citations in the purpose and need section compared to resource analysis sections, meaning that we don’t think that we may have missed much by not looking at the purpose and need. Further, BLM was interested in knowing about the citations used for individual resources, which was another way that we determined that we would focus on resource analysis sections.

As to the appendices, we wanted to focus on the core information within an EA, which all authors agreed would be in the resource analysis sections. We also thought that the resource analysis sections should contain a summary of any analyses in the appendices. While the appendices provide supporting information that is used to support decisions, appendices are not technically considered part of EAs or EISs as the U.S. Council on Environmental Quality, the federal body tasked with overseeing the National Environmental Policy Act (NEPA), does not consider them to count toward the page count of an EA or EIS. Not looking at citations in appendices was a limitation of our study and investigating citations in the appendices could be an area for further investigation in the future.

6) The authors analyze 10 EAs from several proposed action categories. What is the percentage of total EAs by proposed actions? I ask because I’m trying to get a better sense of generalizability.

*Response*: Thanks for this question. We did not include a figure of the total EAs by proposed action in this paper as it is included in another paper of ours that used the same set of EAs that laid a framework for prioritizing science to inform public lands decisions. The distribution of total EAs by proposed action for Colorado and the U.S. for 2015-2019 (the time frame of this paper) can be found as an inset in Figure 1 in the following paper:

Carter, S. K., Haby, T. S., Meineke, J. M., Foster, A. C., McCall, L. E., Espy, L. D., Gilbert, M. A., Herrick, J. E., and K. L. Prentice (2023). Prioritizing science efforts to inform decision making on public lands. Frontiers in Ecology and the Environment. 21(10): 453–460. <https://doi.org/10.1002/fee.2672>

Additionally, the distribution of proposed action categories in our sample of EAs is similar to the distribution of EAs nationally, which is stated on lines 144-146 of the methods section. We added a reference to the figure in Carter et al. 2023, *Frontiers in Ecology and the Environment,* in this sentence to direct readers to this figure should they be curious about the distribution of proposed actions.

7) The table laying out the types of citations is both impressive and a contribution! As is the decision tree/codebook in the supplemental material. Do the authors envision this codebook to be used to analyze citations in the BLM more broadly or other agencies? I suggest the authors talk their typology up a bit more in the conclusion.

*Response*: Thank you for the complements on Table 1 and the decision-tree! Yes, when we set about creating the decision tree we thought that the tool was something that could be used by others working in decision-making using NEPA or a similar framework. When working with BLM staff on another related project we heard BLM staff and others express interest in knowing what types of documents were being cited in NEPA analyses to understand what type of information was making its way to decision makers, what information may be available but is not being used in decisions, and what type of information are readily available and being cited frequently. The decision tree was essential in allowing us to answer these questions, but took substantial time and thought to produce. Answering these questions most completely will require additional analysis in other states and across time to gain a better understanding and we hope that the decision tool will prove to be a helpful tool to others to collect data and gain a better sense of citations in resource analyses.

Thank you for suggesting that we talk up our typology in the conclusion. We have added the following two sentences (lines 526-532 and copied below) to discuss how others may use these methods in the future:

“The decision-tree and document categories that we developed in this study (Supplemental Material Item 1 and Table 1) were developed with the intention that they may be used by others interested in understanding what types of documents are cited in resource analyses. Our study serves as a thorough investigation of citations in EAs completed by the BLM in Colorado over a five-year time period, but applying these methods to another location or time period would provide a more comprehensive and insightful look into citations in resource analyses.”

8) Was intercoder reliability checked to ensure coding concepts were clear and consistent?

*Response*: All the coding was completed by one of the authors who checked in frequently with other authors and other BLM staff to ensure coding accuracy and consistency. Before coding all EAs, the primary coder checked for intercoder reliability by test coding a subsample of EAs with four USGS scientists to assess coding consistency. We have modified the sentence in the methods section (lines 202-205 and copied below) that further describes our coding process and explains our process for testing for intercoder reliability. That revised sentence is copied below:

“One researcher coded all EAs after a period of practice sessions and a test of intercoder reliability where five USGS scientists, including the primary coder, test coded a subsample of EAs to ensure repeatability and coding consistency.”

9) I get a little lost, in multiple places, between the measures on proposed actions versus resources. The section titles are similar, the figures in the supplementary materials only address resources, and so on. I do not have a strong suggestion for improvement (and apologize for offering critique without assist), but think the authors should spend more time thinking through how to discuss and visualize their analyses across these two dimensions.
a. As an example, the authors write that recreation had the oldest literature (roughly line 281) in one section and the newest literature (roughly line 318) in another section.
b. Along these lines, the authors may consider some re-organization, for example presenting results and discussion on proposed actions and then on resources, if those distinctions remain the most important.
c. Relatedly, I am a bit confused on the use of and outcomes of the statistical tests (page 13 especially). There seems to be a lot of exclusions applied. (And statements that seem at odds with one another, such as the note that office was not significant and thus removed and in the results section that it was significant). On the whole, I wonder how worthwhile the use of statistical tests is in a largely descriptive analysis. If the authors feel the need to test for difference, which I think is fine if the n is large enough, then the next version of the paper should make the approach and outcomes clearer. Perhaps a visual demonstrating the statistical comparisons and data subsetting would help?

*Response:* We understand the potential confusion between actions and resources, and this confusion is an issue that we have been working with in both this manuscript and others. We have spent substantial time considering this issue and the current display of information is the result of that consideration. As we have not been able to arrive at an alternative and the reviewer did not provide a potential suggestion we have maintained the current structure of these sections.

As to the comment about recreation actions and recreation resources, we understand the confusion as these are different variables but are both assigned the same name. As a note, we change the name of recreation actions to recreation and visitor services to differentiate the categories. We checked the sections that the reviewer mentioned and the information in both those sections is correct. Recreation and visitor services actions had the highest percentage of older citations (27% of recreation and visitor services action citations were from 1999 or earlier), however sections analyzing potential impacts to recreation as a resource had one of the highest percentages of recent citations (90% of recreation resource citations were from 2010 or later).

Thank you for highlighting potential confusion around the description of statistical tests in the methods section. We agree that this section warranted clarification and have revised this section to improve comprehension. To clarify, office was significant in both of our tests, as was the interaction between office and resources in the second test. However, the interaction between action and office was not significant and this interaction was removed from the first test.

10) I’m not sure all the suppl. figures on resources are needed in a peer-review publication, although I respect that they are needed by BLM partners.

*Response:* Thank you for this comment. We agree that not all the supplemental figures may be needed, however for transparency we wanted to provide the full set of figures. We have alphabetized and created a table of contents for the figures in hopes that readers can easily navigate to the figures that they are interested in.

11) In line 348, the authors write that most citations were categorized as “science” but I’ve forgotten at this point what “science” means in terms of the categories.

*Response:* Thanks for highlighting that we should need to provide clarification on what we placed in the science category. We added a list of the main types of documents that we placed into the science category in parentheses on this line (now line 377). The revised sentence is copied below:

“Most citations were categorized as science (e.g., journal articles, reports, books, theses, conference proceedings) or data (73%), and about half were recent (47% were from 2010 or later).”

12) I was surprised to read near the end that unique references weren’t being counted. I suggest the authors focus on unique references more strongly, perhaps in comparison to citations (repeated references).

*Response:* Thank you for this comment. We agree with the reviewer that it would be valuable to be able to speak to the unique references. Unfortunately, we are unable to speak to unique references for our entire dataset due to our data collection methods as we tallied references within each of our categories. We suggest that recording unique instances of each citation would be an area for improvement in our methods. We re-sampled citations for one resource (noise) at the request of BLM so that we may take an in-depth look at noise as BLM was particularly curious about the noise citations. To take this in depth look, we had to resample all the noise analysis sections and unfortunately it is beyond the scope of this paper to repeat this in-depth look for all. We also want to raise the point that a single reference may be cited in different sections for different resources and that multiple uses of a single reference may not be a true “repeat” of the reference.

13) In lines 411-414, the authors discuss explanations for a small number of core citations. They may also want to consider the likelihood that EA text and references are being recycled. See for example Hileman et al. (2021).

*Response*: Thank you for this suggestion and the citation suggestion. We agree that it is possible that text from EAs and associated citations may have been recycled from one document to the next. We have added a statement to this effect and referenced Hileman et al. (2021) in this section.

14) The authors have some particularly important findings on the representation Indigenous values and interests in the EAs. I think the authors should emphasize these more clearly. Specifically, the analysis reveals how few cites (if any!) are listed in resources addressing Indigenous issues. The authors discuss explanations in lines 421-430. While some of the rationales there are sensible, I want to offer a couple other perspectives. One is that despite there being less peer-reviewed work on on some of these subjects, there is some. TEK has a large literature, for example. Perhaps NEPA authors aren’t consulting the peer reviewed literature in Indigenous studies and other areas. This would be consistent with the evidence I’ve seen that social sciences are underrepresented in NEPA analyses. Another perspective is that the lack of citations reflect a deep engagement with Indigenous people. Although the NEPA analyses pay considerable attention to the issue, the analysis of resource impacts may be more performative.

*Response:* Thank you for this thoughtful comment and discussion! We appreciate the review’s perspective and agree with many of the authors suggestions – there are may be fewer citations to indigenous knowledge as NEPA authors may not be consulting peer reviewed knowledge about indigenous studies, fewer citations may be the result of a general underrepresentation in social sciences, or citations may be unneeded if BLM offices have deep engagement with indigenous peoples. However, we are generally hesitant to speculate about the reasons for a certain type of citations, and specifically to speculate about indigenous knowledge given that our study pre-dated federal direction to consider indigenous knowledge. We did include further discussion on the topic of a lack of citations in the native peoples’ resource analysis sections and state that these sections may have been used to discuss tribal consultation rather analyze of native or indigenous concerns (lines 460-463 and copied below), as our of anecdotal memory from reading these sections was that they tended to focus on tribal consultation.

“Further, while native peoples’ resource analysis sections were formatted as a resource analysis section, the content of these sections often discussed tribal consultation efforts and did not analyze potential effects to indigenous or native peoples’ resources.”

15) I would like to know a little bit more about the BLM offices in Colorado, and the extent to which we can learn how much they differ in context and citation metrics.

*Response:* We considered reporting our results by BLM office to provide greater detail. However, reporting information in this way may be interpreted as a comparison of the quality of resource analysis sections completed by different BLM offices. We did not want evaluate BLM offices individually as this is a sensitive topic for BLM. The agency is required to use science in its decision-making and the BLM is often litigated on this topic. Additionally, it’s possible that authors may use science in their analyses without citing science. Citations are not reflective of all science and data used in decisions and we did not want to provide information that could be attributed to a single office or that could be perceived as an evaluation or comparison of BLM field offices.

Minor:

1) Change parentheses in line 8 to “The Biden administration…”

*Response:* Change made.
2) Line 112, what is “clear science use”?

*Response*: Changed to “explicit use of science”


References

Jacob D. Hileman, Mario Angst, Tyler A. Scott, Emma Sundström. 2021. Recycled text and risk communication in natural gas pipeline environmental impact assessments. Energy Policy 156.

Reviewer #2: This is an interesting paper on an important topic – the use of science in policy decisions. It analyses the citation of scientific and data documents in environmental assessments in a particular policy context and in a particular location.
The work appears to have been thorough and competent. The paper is well written and presented.
Although the analysis is simple in it mathematical/statistical approach, it is appropriate for the research question being addressed.
The paper fills a research gap and potentially establishes a foundation for future research in this area.
I found only one minor typo. Page 9, line 197, change “use” to “used”.

*Response:* Thank you for your positive and succinct review. We have not changed the tense of the word “use” on line 197 as suggested as the current use is correct. The use of the word “use” is speaking to current and ongoing use, not use in the past, so we believe that “use” is grammatically correct.

6. PLOS authors have the option to publish the peer review history of their article ([what does this mean?](https://gcc02.safelinks.protection.outlook.com/?url=https%3A%2F%2Fjournals.plos.org%2Fplosone%2Fs%2Feditorial-and-peer-review-process%23loc-peer-review-history&data=05%7C02%7Calison.foster%40usda.gov%7C02aa679c015e40624f5408dca2eb5f35%7Ced5b36e701ee4ebc867ee03cfa0d4697%7C1%7C0%7C638564379573357185%7CUnknown%7CTWFpbGZsb3d8eyJWIjoiMC4wLjAwMDAiLCJQIjoiV2luMzIiLCJBTiI6Ik1haWwiLCJXVCI6Mn0%3D%7C0%7C%7C%7C&sdata=cf3Oz6b16T48X31crdOmV5s4%2BOsX1Wh%2F%2FDyj8gMkKJ4%3D&reserved=0)). If published, this will include your full peer review and any attached files.

If you choose “no”, your identity will remain anonymous but your review may still be made public.


**Do you want your identity to be public for this peer review?** For information about this choice, including consent withdrawal, please see our [Privacy Policy](https://gcc02.safelinks.protection.outlook.com/?url=https%3A%2F%2Fwww.plos.org%2Fprivacy-policy&data=05%7C02%7Calison.foster%40usda.gov%7C02aa679c015e40624f5408dca2eb5f35%7Ced5b36e701ee4ebc867ee03cfa0d4697%7C1%7C0%7C638564379573363309%7CUnknown%7CTWFpbGZsb3d8eyJWIjoiMC4wLjAwMDAiLCJQIjoiV2luMzIiLCJBTiI6Ik1haWwiLCJXVCI6Mn0%3D%7C0%7C%7C%7C&sdata=QKKK6%2BsrxrEQfmljWKql%2FdyJ8lMl9ocwPt1Od%2BHDo3Y%3D&reserved=0).

Reviewer #1: No

Reviewer #2: **Yes:** David Pannell

[NOTE: If reviewer comments were submitted as an attachment file, they will be attached to this email and accessible via the submission site. Please log into your account, locate the manuscript record, and check for the action link "View Attachments". If this link does not appear, there are no attachment files.]

While revising your submission, please upload your figure files to the Preflight Analysis and Conversion Engine (PACE) digital diagnostic tool, [https://pacev2.apexcovantage.com/](https://gcc02.safelinks.protection.outlook.com/?url=https%3A%2F%2Fpacev2.apexcovantage.com%2F&data=05%7C02%7Calison.foster%40usda.gov%7C02aa679c015e40624f5408dca2eb5f35%7Ced5b36e701ee4ebc867ee03cfa0d4697%7C1%7C0%7C638564379573368804%7CUnknown%7CTWFpbGZsb3d8eyJWIjoiMC4wLjAwMDAiLCJQIjoiV2luMzIiLCJBTiI6Ik1haWwiLCJXVCI6Mn0%3D%7C0%7C%7C%7C&sdata=ig2vDJ5GCYjxbx%2Fli6shj5igd7Zvb7903IcMTHU9JwA%3D&reserved=0). PACE helps ensure that figures meet PLOS requirements. To use PACE, you must first register as a user. Registration is free. Then, login and navigate to the UPLOAD tab, where you will find detailed instructions on how to use the tool. If you encounter any issues or have any questions when using PACE, please email PLOS at [figures@plos.org](mailto:figures@plos.org). Please note that Supporting Information files do not need this step.
